# Optical Manipulations Reveal Strong Reciprocal Inhibition But Limited Recurrent Excitation within Olfactory Bulb Glomeruli

**DOI:** 10.1523/ENEURO.0311-21.2021

**Published:** 2021-12-07

**Authors:** Joseph D. Zak, Nathan E. Schoppa

**Affiliations:** 1 Neuroscience Program, University of Colorado Anschutz Medical Campus, Aurora, Colorado 80045; 2 Department of Physiology and Biophysics, University of Colorado Anschutz Medical Campus, Aurora, Colorado 80045

**Keywords:** circuits, glomerulus, olfaction, olfactory bulb, optogenetics, synapses

## Abstract

The local circuitry within olfactory bulb (OB) glomeruli filters, transforms, and facilitates information transfer from olfactory sensory neurons to bulb output neurons. Two key elements of this circuit are glutamatergic tufted cells (TCs) and GABAergic periglomerular (PG) cells, both of which actively shape mitral cell activity and bulb output. A subtype of TCs, the external TCs (eTCs), can synaptically excite PG cells, but there are unresolved questions about other aspects of the glomerular connections, including the extent of connectivity between eTCs and the precise nature of reciprocal interactions between TCs and PG cells. We combined patch-clamp recordings in OB slices and optophysiological tools to investigate local functional connections within glomeruli of mice and rats. When TCs that express cholecystokinin (CCK) were optically suppressed, excitatory inputs to “uniglomerular” PG cells that extend dendrites to one glomerulus were decreased, consistent with TC activation being required for most excitation of these PG cells. However, TC suppression had no effect on EPSCs in eTCs, arguing that TCs make few, if any, direct glutamatergic synaptic connections with eTCs. The absence of synaptic connections among eTCs was also supported by recordings in eTC pairs. Last, we show using similar optical suppression methods that GAD65-expressing PG cells, mainly uniglomerular cells, provide strong inhibition in eTCs. Our results imply that the local network of CCK-expressing TCs form potent reciprocal chemical synaptic connections with GAD65-expressing uniglomerular PG cells but not eTCs. This configuration favors local inhibition over recurrent excitation within a glomerulus, limiting its output.

## Significance Statement

The brain circuits involved with the initial processing of sensory information are composed of networks of excitatory and inhibitory neurons. The strength of local connections can impact the weighting of excitation versus inhibition and determine whether sensory signals are passed on to higher-order brain structures. We investigated the connectivity between excitatory and inhibitory neurons in discrete glomerular structures of the rodent olfactory bulb. We found that excitatory neurons make potent chemical synaptic connections with a class of inhibitory cells with dendrites confined to one glomerulus. However, excitatory cells do not form chemical synaptic connections with each other. This circuit configuration suggests that sensory input is generally biased toward driving more local inhibition than recurrent excitation, thus suppressing bulbar output.

## Introduction

Olfactory bulb (OB) glomeruli not only serve as a relay between the sensory periphery and the olfactory cortex but are also the site of initial information processing in the olfactory system. Glomeruli are innervated by multiple cell types that facilitate the detection and discrimination of odors through interconnected networks of excitatory and inhibitory neurons (Nagayama et al., 2014; Takahashi et al., 2016; Burton, 2017). A class of excitatory interneurons known as external tufted cells (eTCs) are a central element that controls both excitation and inhibition within glomeruli (Hayar et al., 2004b; De Saint Jan et al., 2009; Gire and Schoppa, 2009; Najac et al., 2011; Gire et al., 2012, 2019). For example, eTCs can directly excite a subset of GABAergic periglomerular (PG) cells through glutamatergic synaptic contacts (Hayar et al., 2004b). These PG cells, which have functionally been defined as eTC-driven (ETd) cells (Shao et al., 2009), account for ∼70% of the total number of PG cells and are likely to predominately correspond to histologically defined type II PG cells (Kosaka et al., 1996). At the same time, eTCs also excite OB output mitral cells (MCs; De Saint Jan et al., 2009; Najac et al., 2011; Gire et al., 2012). This eTC-to-MC excitation, which appears to result from glutamate “spillover” at eTC-to-PG cell dendrodendritic synapses (Gire et al., 2019), accounts for the majority of the excitatory charge in MCs following the stimulation of olfactory sensory neurons (OSNs; Gire et al., 2012; Vaaga and Westbrook, 2016). Between glomeruli, laterally projecting short-axon cells bidirectionally modulate activity at neighboring glomeruli through neurotransmitter corelease and electrical coupling (Aungst et al., 2003; Maher and Westbrook, 2008; Liu et al., 2013, 2016; Banerjee et al., 2015).

Despite progress in understanding the functional connectivity of glomerular neurons, there remain numerous unanswered questions. For example, while eTCs can clearly excite ETd-PG cells, their connectivity to other eTCs is not well resolved. Because of the high sensitivity of eTCs to OSN input (De Saint Jan et al., 2009; Gire et al., 2012), eTC-to-eTC excitation could mediate an important type of recurrent excitation within a glomerulus following OSN stimulation that could impact output neuron activity. Ultrastructural studies have provided somewhat differing perspectives on whether there are dendrodendritic excitatory synapses within glomeruli (Kosaka and Kosaka, 2005; Bourne and Schoppa, 2017), potential eTC-to-eTC contacts, and whether eTCs form synapses with each other has not been well examined using physiological methods. The role of eTCs in exciting PG cells is also not completely understood, especially as it relates to how much eTCs contribute to excitation of ETd-PG cells versus other sources. For example, PG cells can be excited directly by MCs (Najac et al., 2015). The last point pertains to the connections from PG cells back onto eTCs. Prior studies have established that different subtypes of morphologically distinct PG cells can be defined by the isoform of glutamic acid decarboxylase (GAD) that they express (Parrish-Aungst et al., 2007; Kiyokage et al., 2010), either GAD65 or GAD67. It is not clear which of these subtypes provides most of the inhibition of eTCs.

We studied the local functional connectivity within glomeruli, first, using an optogenetic silencing approach in cholecystokinin (CCK)-Cre mice. CCK is highly expressed in OB TCs of different subtypes, including eTCs (Sun et al., 2020), and thus these mice provided a convenient tool to examine the functional connectivity from TCs to different cell types. Second, we used dual whole-cell recordings that included an eTC and either another eTC or a PG cell. These experiments provided evidence that both CCK-expressing TCs in general, as well as eTCs in particular, in fact, do not form direct excitatory synaptic connections with other eTCs, although they potently excite PG cells. Also, activation of CCK-expressing neurons underlies most excitation of ETd-PG cells. As a final element of our study, we used recordings in GAD65-Cre mice to provide evidence that GAD65-expressing PG cells mediate a major source of inhibition onto eTCs. Together, our findings support a model in which the local network of TCs form potent reciprocal connections with GAD65-expressing GABAergic interneurons but not eTCs.

## Materials and Methods

### Animals

CCK-Cre mice (Chhatwal et al., 2007; stock #011086, The Jackson Laboratory) were maintained as heterozygous breeding pairs. Heterozygous CCK-Cre offspring were then crossed with LSL-eNpHR3.0-EYFP mice (Madisen et al., 2012; stock #014539, The Jackson Laboratory). The resulting male and female offspring were used for optogenetic experiments. CCK-Cre mice were also crossed with LSL-tdTomato mice (Madisen et al., 2010; stock #07909, The Jackson Laboratory) to generate reporter animals to verify CCK expression. GAD65-Cre mice (Taniguchi et al., 2011; stock #019022, The Jackson Laboratory) were also maintained as heterozygous breeding pairs and crossed with LSL-eNpHR3.0-EYFP or LSL-ChR2-EYFP mice (Madisen et al., 2012). GAD65-Cre mice were also crossed with LSL-tdTomato mice to make reporter animals. Male and female Sprague Dawley rats at postnatal day 8 (P8) to P20 (Charles River Laboratories) were used for the pair-cell recordings. Rats were used for these studies because of the low yield of the pair-cell recordings, which required a large number of test animals. Rats were readily available in the Schoppa laboratory at the time that the pair-cell recordings were performed. All animals were housed on a 12 h light/dark schedule and fed *ad libitum*. All experiments were conducted under protocols approved by the Animal Care and Use Committee of the University of Colorado, Anschutz Medical Campus.

### Slice preparation

Acute horizontal OB slices (300–330 μm) were prepared following isoflurane anesthesia and decapitation. For experiments using genetic expression of light-gated ion channels, adult mice (age, >6 weeks) were used to allow for sufficient channel expression. Both OBs were rapidly removed and placed in oxygenated (95% O_2_, 5% CO_2_) ice-cold solution containing the following (in mm): 72 sucrose, 83 NaCl, 26 NaHCO_3_, 10 glucose, 1.25 NaH_2_PO_2_, 3.5 KCl, 3 MgCl_2_, and 0.5 CaCl_2_ adjusted to 295 mOsm. OBs were separated into hemispheres with a razor blade and attached to a stage using adhesive glue (Loctite 404, Henkel) applied to the ventral surface of the tissue. Slices were cut using a vibrating microtome (model VT1000S, Leica) and were incubated in a holding chamber for 30 min at 32°C. Subsequently, the slices were stored at room temperature.

### Electrophysiology

All electrophysiology experiments were conducted under an upright Zeiss Axioskop2 FS Plus microscope (Carl Zeiss MicroImaging) fitted with differential interference contrast optics, video microscopy under control of Slidebook software (3i), and a CCD camera (Hamamatsu). Cells were visualized and identified with 10× or 40× Zeiss water-immersion objectives. All recordings were performed at 32–35°C.

The base extracellular recording solution contained the following (in mm): 125 NaCl, 25 NaHCO_3_, 1.25 NaHP0_4_O, 25 glucose, three KCl, 1 MgCl_2_, and 2 CaCl_2_, at pH 7.3 and adjusted to 295 mOsm, and was oxygenated (95% O_2_, 5% CO_2_). The base intracellular pipette solution for whole-cell current-clamp recordings contained the following (in mm): 125 K-gluconate, 2 MgCl_2_, 0.025 CaCl_2_, 1 EGTA, 2 Na_3_ATP, 0.5 Na_3_GTP, and 10 HEPES, at pH 7.3 with KOH, and osmolarity adjusted to 215 mOsm (Zak et al., 2015). For some current recordings, K-gluconate in the pipette solution was replaced with an equimolar amount of cesium methanosulfonate, as well as the sodium channel blocker QX-314 (10 mm) to block action potentials. In all whole-cell current-clamp recordings from eTCs, 30 mm glutamic acid was added to the pipette to prevent the rundown of evoked neurotransmitter release (Ma and Lowe, 2007). Loose cell-attached (LCA) recordings from eTCs were made with a pipette that contained 150 mm NaCl. All whole-cell recordings included 100 μm Alexa Fluor 488 or Alexa Fluor 594 (Thermo Fisher Scientific) in the pipette solution to allow for the visualization of cell processes. Fluorescence measurements were performed under whole-field epi-illumination using a DG-4 light source (Sutter Instrument). Signals were detected by a Cool-Snap II HQ CCD camera (Photometrics) under the control of Slidebook software.

Borosilicate glass patch pipettes (World Precision Instruments) were pulled to a resistance of 4–6 MΩ for eTCs, 6–8 MΩ for PG cells, and 3–4 MΩ for MCs using an upright puller (Narishige). Current and voltage signals in the single-cell and pair-cell experiments were recorded with a Multiclamp 700B amplifier (Molecular Devices), low-pass filtered at 1.8 kHz using an eight-pole Bessel filter and digitized at 10 kHz using a Digidata 1322A (Molecular Devices) digital interface. Data were acquired using AxographX software on an Apple MacPro computer.

Cell identity was determined in part by visualizing Alexa Fluor 488-mediated or Alexa Fluor 594-mediated fluorescence signals. eTCs were distinguished from PG cells by their position in the inner half of the glomerular layer, their relatively large, spindle-shaped somata (diameter, ∼10 μm), a single highly branched apical dendrite, lack of lateral dendrites, and relatively low input resistance (∼0.2 GΩ; Hayar et al., 2004a). PG cells were identified by their small soma (diameter, <10 μm) and high input resistance (>0.8 GΩ; Hayar et al., 2004a; Murphy et al., 2005; Shao et al., 2009). Test PG cells were all uniglomerular, with an apical dendrite that extended to just one glomerulus (Kiyokage et al., 2010). We only considered PG cells that displayed bursts of EPSCs either spontaneously or in response to OSN stimulation, consistent with ETd cells (Kosaka et al., 1998; Shao et al., 2009). MCs were easily identified by their position in the mitral cell layer and distinctive dendritic arborizations.

In the pair-cell recordings, cell-fill images were used to confirm that the primary dendrite of both neurons occupied the same glomerular neuropil. Visual inspections were further confirmed by measurements of excitatory charge transfer on stimulation of one of the cells. In 5 of 5 eTC–PG cell pairs and 22 of 25 eTC–eTC pairs, stimulation resulted in excitation from one cell to the other, substantiating their joint association with the same glomerulus. In the case of the eTC–eTC pairs, the observed charge transfer was kinetically slow.

Stimulation of OSN axons was performed using a broken-tip patch pipette (diameter, 5–10 μm) placed in the olfactory nerve layer, ∼50–100 μm superficial to the glomerular layer. Current injections were delivered by a stimulus isolator (World Precision Instruments) under control of a transistor–transistor logic (TTL) output from AxographX software. Weak intensities of electrical stimulation were used (5–50 μA). eTCs or PG cells that were chosen were associated with glomeruli at the surface of the slice. Stimulus artifacts in many of the illustrated traces have been blanked or truncated. Optogenetic stimulation of NpHR3.0 was performed using a 140 mW, 590 nm collimated LED (ThorLabs) with epifield illumination through a 40× objective under control of a TTL output from AxographX software. Channelrhodopsin-2 (ChR2) stimulation was performed using a 760 mW, 470 nm LED (ThorLabs). For displayed voltage-clamp traces of eTCs where NpHR3.0 is activated (see Fig. 2*D*), current artifacts were removed by subtracting a median filtered signal from each trial individually.

### Imaging

Confocal images of CCK-tdTomato and GAD65-tdTomato expression were acquired by scanning laser confocal microscope (Fluoview FV1000, Olympus). Maximum projection images were generated from *z*-stacks at 1 μm intervals in a single field of view using Fiji (Schindelin et al., 2012).

### Experimental design and statistical methods

Evoked and spontaneous EPSCs (sEPSCs) were detected using a variable amplitude template with a 0.5 ms rise and 2 ms decay, consistent with typical AMPA receptor kinetics (Schoppa, 2006; Tyler et al., 2007; Grubb et al., 2008). EPSCs with amplitudes <2.5 SDs of baseline noise were rejected. Data were analyzed using AxographX and custom MATLAB scripts (MathWorks). Data throughout are expressed as the mean ± SEM.

Peristimulus time histograms (PSTHs) of EPSC frequency and spike rates were computed by calculating the mean frequency in 20–50 ms time bins from ≥10 sweeps. Significance was determined using two-tailed nonparametric tests, a Wilcoxon signed-rank test for matched pairs, or a Wilcoxon rank-sum test for nonmatched samples. Paired *t* tests were used in some matched-pair comparisons because of a smaller sample size when *n *<* *6. Throughout, a value of *p *<* *0.05 was considered significant.

## Results

### Optical suppression of CCK-expressing cells

**Figure 1.**
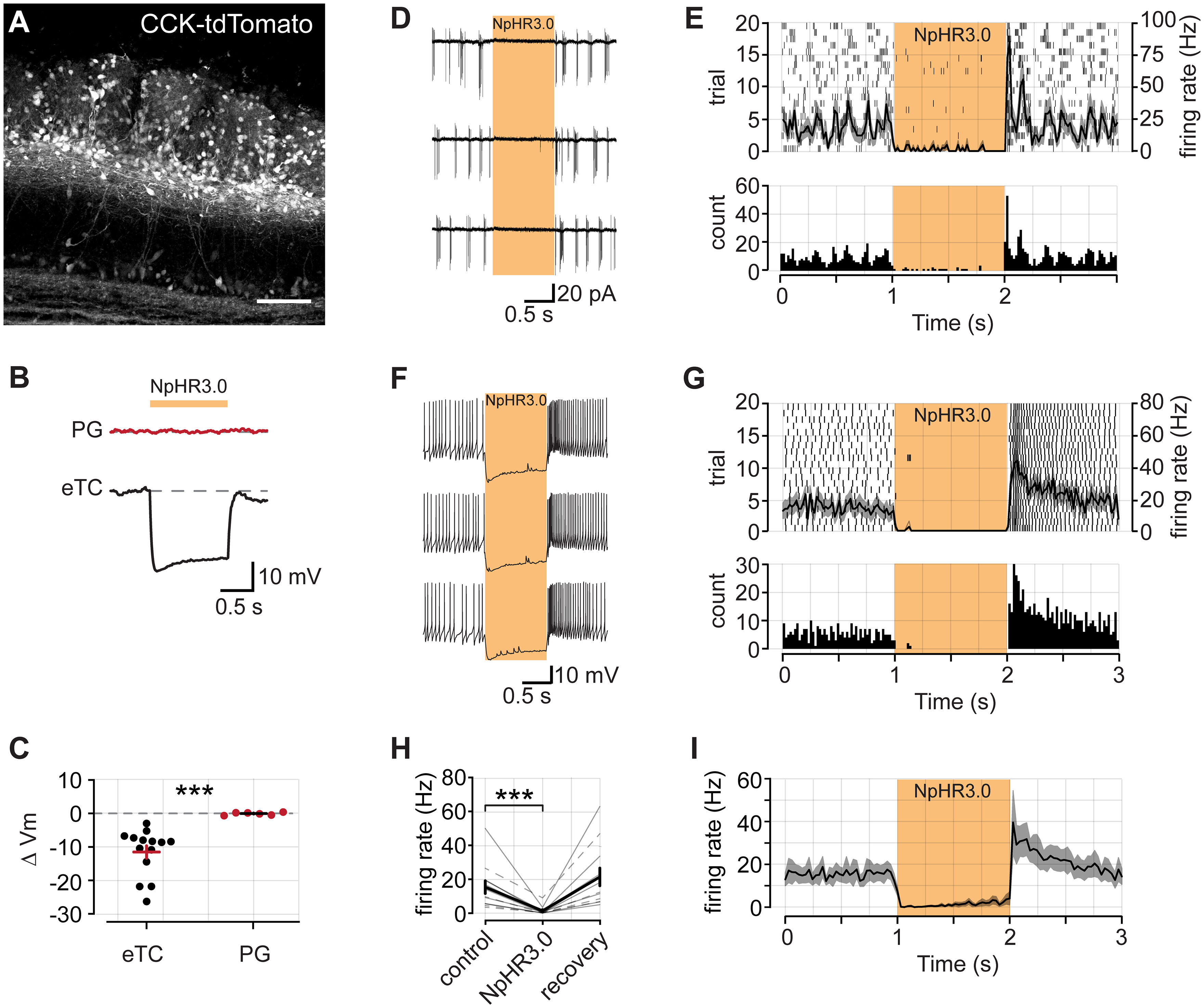
CCK-Cre mice provide a tool to suppress activity specifically in bulbar TCs. ***A***, CCK expression in a tdTomato reporter mouse line. CCK is densely expressed in cells in the inner portion of the glomerular layer and outer portion of the external plexiform layer. Scale bar, 100 μm. ***B***, Example membrane voltage responses from a PG cell (top) and an eTC (bottom) in a CCK-NpHR3.0 mouse in response to a 1 s pulse of 590 nm LED light. ***C***, Summary data of NpHR3.0-induced membrane voltage changes in eTCs (*n* = 14) and PG cells (*n* = 6). ***D***, Example spike recordings from an eTC in LCA configuration. Yellow bar denotes a 590 nm light pulse. ***E***, Top, Raster plot of 20 trials recorded from the same cell as in ***D***. The mean PSTH of all trials (black curve) is superimposed. Shaded area denotes SE. Bin width = 30 ms. Bottom, A histogram of all trials. ***F***, ***G***, Example spike recordings from an eTC in current-clamp configuration. Plots are the same as in ***D*** and ***E***. ***H***, Summary data from combined LCA (*n *=* *4; dashed lines) and current-clamp (*n* = 8; solid lines) experiments. Firing rate calculated as the mean in each epoch. Error bars indicate the SEM. ***I***, Mean PSTH across all recordings (*n* = 12). *** denotes *p* < 0.001.

In the OB, the peptide hormone CCK is selectively expressed in TCs (including eTCs), but not in MCs (Seroogy et al., 1985; Economo et al., 2016; Sun et al., 2020). We first confirmed CCK expression and spatial distributions by generating CCK-tdTomato mice (see Materials and Methods; Chhatwal et al., 2007; Madisen et al., 2012). In OB slices from reporter mice, tdTomato expression was largely confined to the inner half of the glomerular layer and outer portion of the external plexiform layer (EPL; Fig. 1*A*), consistent with prior descriptions of the spatial distribution of CCK expression (Sun et al., 2020). These TCs included eTCs, which reside in the glomerular layer and lack lateral dendrites, and superficial TCs (sTCs) that have lateral dendrites that extend just below the glomerular layer through the external plexiform layer. We also observed sparse labeling of cells deeper in the bulb, which are likely middle and deep TCs. We then took advantage of this selective expression by crossing CCK-Cre mice with a conditional line for the light-activated chloride pump halorhodopsin (NpHR3.0) to generate CCK-NpHR3.0 animals. These mice allowed us to test how optical suppression of TCs influences excitatory signaling within OB glomeruli. Because we could not differentiate between subtypes of TCs based on CCK expression alone, we refer to the impacted cells on optogenetic suppression collectively as TCs.

To test the efficacy of NpHR3.0 expression, we recorded from visually identified eTCs or PG cells in the glomerular layer of the OB (see Materials and Methods). In current-clamp configuration, after a 1 s baseline period, we delivered a 1 s pulse of 590 nm light to the slice to activate NpHR3.0 (Fig. 1*B*,*D*,*F*). eTCs were strongly hyperpolarized by illumination (−11.39 ± 1.87 mV; *n *=* *14), while there was no effect on PG cell membrane potential (0.10 ± 0.15 mV change; *n *=* *6; *p *<* *0.001 comparison to eTCs, Wilcoxon rank-sum test; Fig. 1*B*,*C*). Also, in three MCs tested, we did not observe significant light-evoked hyperpolarizations (−1.24 ± 0.64 mV; *n = 3*). In one of the MCs, there was a small hyperpolarization (−2.1 mV), but this may have reflected weak electrical coupling between CCK-expressing eTCs and MCs (De Saint Jan et al., 2009; Gire et al., 2012). Thus, NpHR3.0 expression appears to be high in TCs but not in other OB cells. In terms of preventing action potential firing in eTCs, the light pulses were highly efficacious. Across 12 eTC recordings, conducted in either whole-cell current-clamp (*n *=* *8) or loose cell-attached patch configurations (*n *=* *4), light nearly eliminated spontaneous spiking (baseline epoch, 15.47 ± 3.80 Hz; vs LED epoch, 1.39 ± 0.75 Hz; *p *=* *0.0005, Wilcoxon signed-rank test; Fig. 1*H*,*I*). In some cells, and shown in the summary data in Figure 1*I*, a small recovery in spiking was observed over the course of the light pulse. This recovery likely stems from a hyperpolarization-activated current that is a hallmark of eTCs (Liu and Shipley, 2008; De Saint Jan et al., 2009), and can be observed in the recordings in Figure 1, *B* and *F*.

### Under baseline conditions, CCK-expressing neurons provide most of the direct excitatory input onto ETd-PG cells while providing no direct input onto eTCs

**Figure 2.**
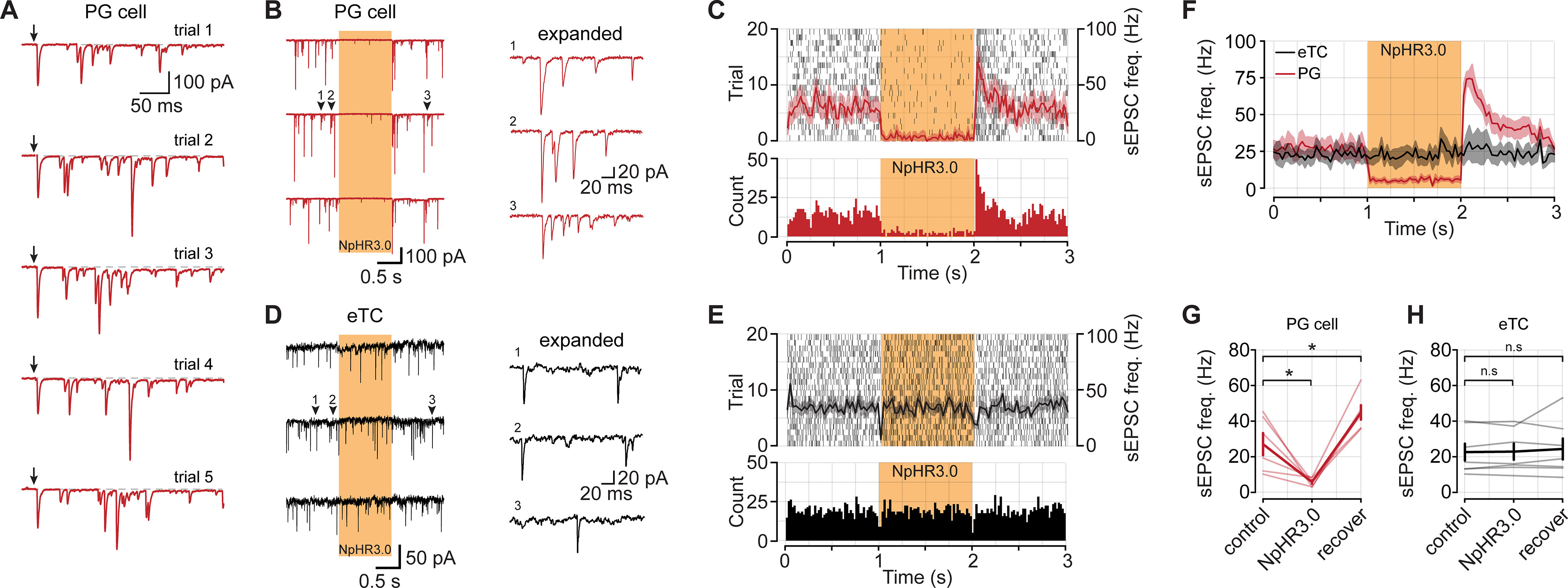
Light application in CCK-NpHR3.0 mice reduces the frequency of spontaneous excitatory synaptic inputs onto PG cells but not eTCs. ***A***, The subtype of PG cell (external tufted driven vs olfactory nerve driven) for this analysis was determined by applying electrical stimulus pulses to OSN axons (downward arrows). Note the barrage of evoked EPSCs in the voltage-clamped cell (*V*_hold_ = –77 mV), characteristic of ETd-PG cells. ***B***, Example recordings of sEPSCs (no stimulation) in an ETd-PG cell across three consecutive sweeps. The yellow bar denotes a 590 nm light pulse for 1 s to suppress TC output. The traces to the right depict expanded sections of the second sweep, highlighting examples of sEPSC bursts that reflect spike bursts in connected eTCs (Shao et al., 2009). ***C***, Top, Raster plot of 20 trials recorded from the same cell as in ***A***. The mean PSTH of all trials is superimposed in red, the shaded area denotes SEM. Bottom, A histogram of all trials from the raster plot above. ***D***, Example sEPSC recordings from an eTC and expanded traces to the right noting the absence of sEPSC bursts. ***E***, Plots are same as ***B***, but for the eTC shown in ***D***. ***F***, Mean sEPSC PSTHs across all PG cells (*n *=* *6) and eTCs (*n *=* *7). ***G***, Summary data from all PG cells. ***H***, Summary data from all eTCs. * denotes *p* < 0.05, n.s. not significant.

With the ability to strongly and selectively suppress TC output, we next examined the contribution of TCs to the excitation of different neurons in the glomerular layer. In this analysis, we focused on both uniglomerular PG cells that extend their dendrites to one glomerulus (Kiyokage et al., 2010), as well as eTCs. Among PG cells, there is a subpopulation of functionally defined ETd-PG cells that receive direct glutamatergic synaptic input from eTCs (Hayar et al., 2004b; Shao et al., 2009), but the contribution of other glutamatergic cells to their excitation is not well resolved. For example, at least some PG cells can be directly excited by MCs (Najac et al., 2015). The CCK-NpHR3.0 mice provided a tool to estimate the relative contribution of CCK-expressing neurons (including eTCs and sTCs) onto excitation of ETd-PG cells versus other sources, based on how much of the excitatory current recorded in ETd-PG cells was eliminated by light. ETd-PG cells were identified in our recordings based on the presence of a prolonged barrage of EPSCs in response to electrical stimulation of OSNs (Fig. 2*A*).

In the initial analysis, we evaluated sEPSCs that were isolated in voltage-clamped PG cells and eTCs (*V*_hold_ = −77 mV) in CCK-NpHR3.0 mice in the absence of stimulation (Fig. 2*B*). In the PG cell recordings, the spontaneous EPSCs often occurred as bursts of events, characteristic of inputs from eTCs that engage in spike bursts (Hayar et al., 2004a; Shao et al., 2009; Fig. 1*D*, example). We found that light application resulted in a large, reversible reduction in the frequency of sEPSCs in PG cells (reduction, 69.65 ± 9.41%; *n *=* *6; *p *=* *0.031; Fig. 2*B*,*C*,*F*,*G*). In contrast, sEPSCs in eTCs were unaffected (increase in frequency, 2.22 ± 3.81%; *n *=* *7; *p *=* *0.689; Fig. 2*D*,*E*,*H*). These data support the idea that CCK-expressing TCs are a primary source of input to ETd-PG cells under baseline conditions but provide little, if any, direct input to eTCs. Interestingly, in the PG cell recordings, the sEPSC rate during the recovery epoch after light application was somewhat higher than the control in the prestimulus period (baseline, 27.07 ± 6.25 Hz; vs recovery, 44.89 ± 4.05 Hz; *p *=* *0.031, Wilcoxon signed-rank test; Fig. 2*F*,*G*). This likely reflected eTC spike bursts that were activated by NpHR3.0-induced hyperpolarization-activated currents (Liu and Shipley, 2008; De Saint Jan et al., 2009).

**Figure 3.**
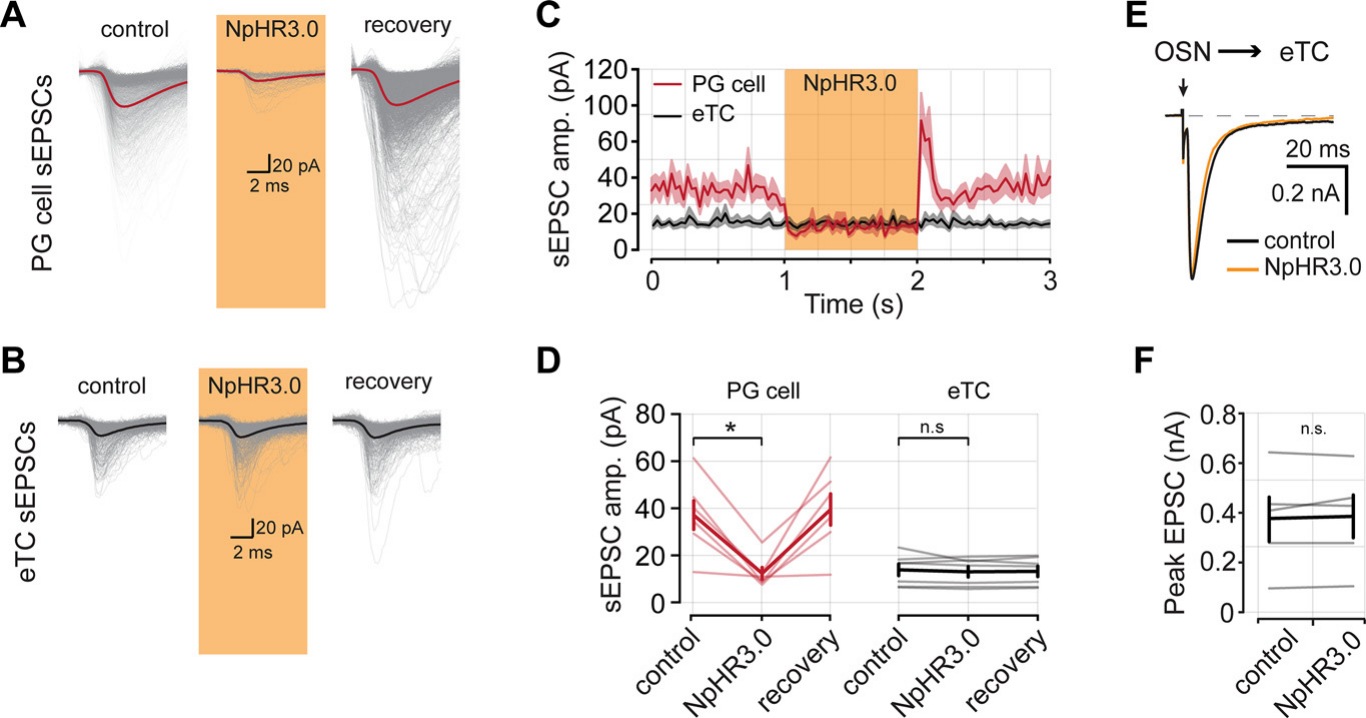
Light application in CCK-NpHR3.0 mice eliminates large-amplitude sEPSCs in PG cells. ***A***, Aligned sEPSCs captured from an example PG cell in each epoch. ***B***, Aligned sEPSCs captured from an example eTC. ***C***, PSTH of mean sEPSC amplitude across all PG cells and eTCs (*n *=* *6 PG cells, *n *=* *7 eTCs). ***D***, Summary data from all cells in each recording epoch eTCs [reduction in PG cells, 61.84 ± 9.87% (*p *=* *0.031); reduction in eTCs cells, 5.18 ± 4.22% (*p *=* *0.688; Wilcoxon signed-rank test)]. ***E***, Example EPSCs evoked by OSN stimulation recorded from an eTC and during TC inactivation with NpHR3.0. Downward arrow denotes stimulus to OSNs. ***F***, Summary of evoked EPSC amplitudes from five eTCs (control trials, 372.31 ± 91.51 pA; vs NpHR3.0 trials, 381.39 ± 88.64 pA; *p *=* *0.945; Wilcoxon signed-rank test). * denotes *p* < 0.05, n.s. not significant.

We also measured the amplitudes of the sEPSCs for both PG cells and eTCs (Fig. 3*A–C*). During light application, the amplitudes of the sEPSCs in ETd-PG cells were drastically reduced (reduction, 61.84 ± 9.87%; *p *=* *0.031, Wilcoxon signed-rank test; Fig. 3*D*). In these recordings, some smaller-amplitude sEPSCs persisted (Fig. 3*A*,*C*,*D*), which may reflect miniature EPSCs (mEPSCs). mEPSCs are thought to arise from action potential-independent release at a single synapse and therefore should be insensitive to light-driven membrane hyperpolarizations in TCs. In contrast to the potent effects of light on sEPSC amplitude in PG cells, light had no effect on the sEPSC amplitudes in eTCs (reduction, 5.18 ± 4.22%; *p *=* *0.688, Wilcoxon signed-rank test; Fig. 3*B–D*). Finally, to demonstrate that NpHR3.0 activation had no effect on release from OSN axon terminals, we measured evoked monosynaptic EPSCs from OSNs to eTCs (Fig. 3*E*). We found no difference in the peak evoked EPSC amplitudes for the same stimulus intensity under control conditions and during slice illumination (control trials, 372.31 ± 91.51 pA; vs NpHR3.0 trials, 381.39 ± 88.64 pA; *n *=* *5; *p *=* *0.945, Wilcoxon signed-rank test; Fig. 3*F*). Overall, our analysis of EPSC amplitudes was consistent with the conclusions from analyzing sEPSC frequency that TCs are a major source of excitatory input onto ET-PG cells under baseline conditions, but they provide little excitatory input onto eTCs (see Discussion).

### Excitation of PG cells driven by OSN stimulation requires activation of TCs

**Figure 4.**
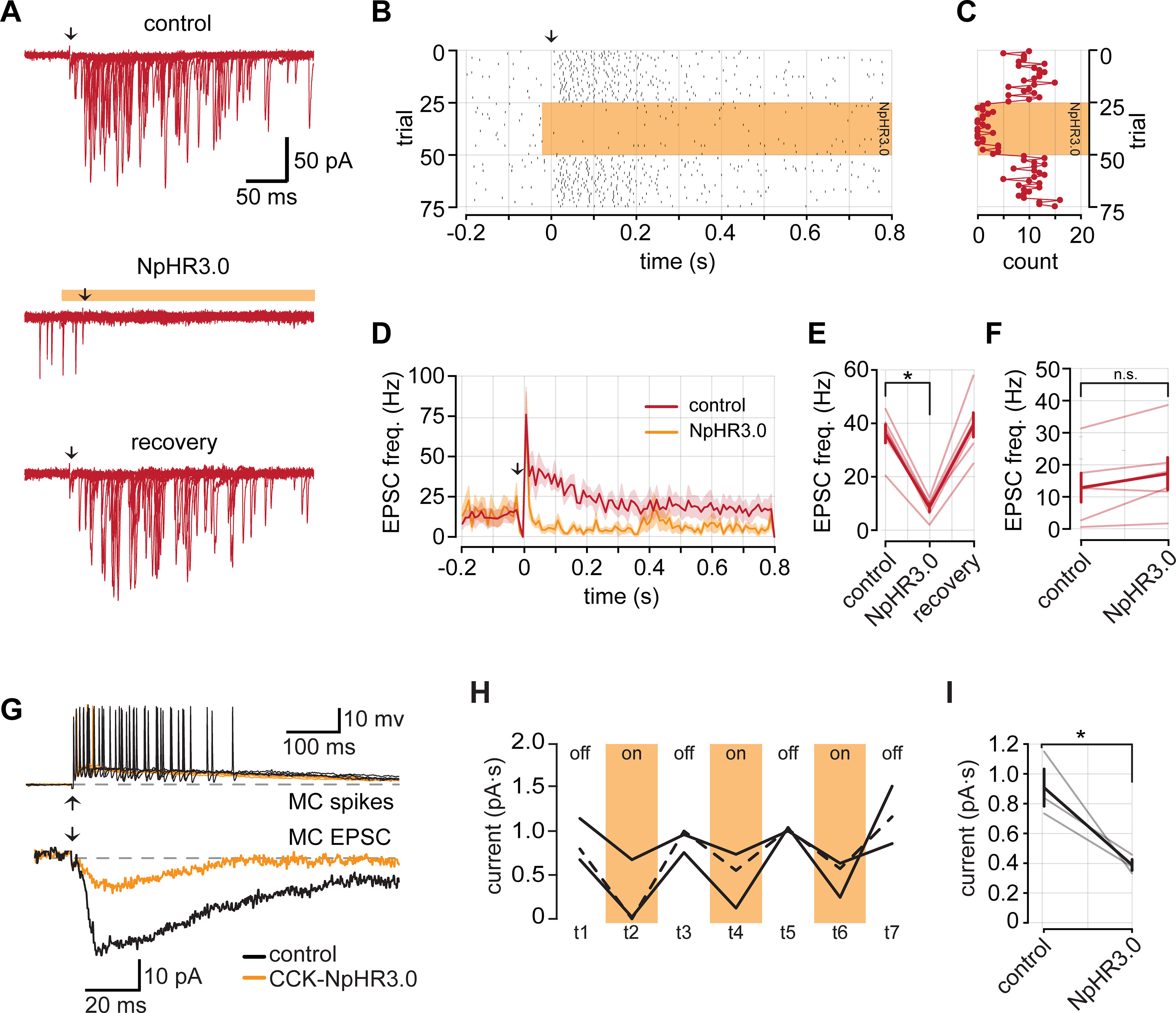
Evoked EPSCs in ETd-PG cells require activation of CCK-expressing TCs. ***A***, Example EPSCs evoked by OSN stimulation in a PG cell. Each panel contains five overlaid trials. Downward arrows correspond to OSN stimulation. ***B***, Raster plot of EPSCs from the PG cell in ***A***. The yellow bar indicates the presence of 590 nm light to suppress CCK-expressing cells with NpHR3.0. ***C***, EPSC count of each trial in ***B*** in the 300 ms immediately following OSN stimulation. ***D***, Summary PSTH of EPSC frequency across all PG cells (*n *=* *6). Red trace, control trials; yellow trace, trials with the LED on. Note: the drop in EPSC frequency right at OSN stimulation reflects obscuring of the EPSCs by the stimulus artifact; the jump in EPSC frequency that follows is consistent with the kinetics of a monosynaptic OSN-driven EPSCs in PG cells that has been reported by Shao et al. (2009). ***E***, Summary data of evoked EPSC frequency in the 10–300 ms window following ON stimulation (control trials, 36.21 ± 3.47 Hz; vs NpHR3.0 trials, 8.46 ± 1.52 Hz; *n *=* *6; *p *=* *0.031, Wilcoxon signed-rank test). ***F***, Summary of baseline EPSC frequency during control and NpHR3.0 trials (control trials, 12.93 ± 4.57 Hz; vs NpHR3.0 trials, 17.31 ± 5.08 Hz; *p *=* *0.094, Wilcoxon signed-rank test). ***G***, Top, Evoked spikes recorded in an MC under control conditions and during TC inactivation. Bottom, Example excitatory currents recorded from the same MC. TC inactivation reduced the size of the current. ***H***, Excitatory current area measurements from three MCs on interleaved trials of TC inactivation with NpHR3.0. The dashed line represents the MC in ***G***. ***I***, Summary data of effects on TC inactivation on OSN-evoked excitatory currents measured in MCs. * denotes *p* < 0.05, n.s. not significant.

In addition to baseline conditions, we also wanted to know the contribution of different mechanisms of exciting PG cells when the OB network is excited by OSN stimulation. The role of eTCs in particular in exciting PG cells may be unusually high under baseline conditions because of the high level of spontaneous spiking in eTCs (Hayar et al., 2004a). In these studies, we recorded from ETd-PG cells and delivered electrical stimulation to OSNs (100 μs; variable intensity) that was sufficient to generate EPSC barrages on every trial (Fig. 4*A*). We then suppressed activity of CCK-expressing neurons using light-evoked activation of NpHR3.0 on alternating blocks of 25 trials (Fig. 4*A–C*); in trials with light pulses, OSN stimulation was applied 20 ms after the start of the light pulses. We found that light caused a large ∼75% reduction in the number of evoked EPSCs in PG cells (control trials, 36.21 ± 3.47 Hz; vs NpHR3.0 trials, 8.46 ± 1.52 Hz; *n *=* *6; *p *=* *0.031, Wilcoxon signed-rank test; Fig. 4*D*,*E*). In three of six PG cells, we observed an early EPSC that was consistent with the kinetics of a monosynaptic OSN-driven EPSC (Shao et al., 2009). We excluded these events from our analysis by using a 10–300 ms window following OSN stimulation. The decrease in evoked EPSCs in PG cells was not the result of changes in the underlying baseline sEPSC rate on control versus NpHR3.0 trials, since these baseline rates were not different (control trials, 12.93 ± 4.57 Hz; vs NpHR3.0 trials, 17.31 ± 5.08 Hz; *p *=* *0.094, Wilcoxon signed-rank test; Fig. 4*F*). Thus, our results provide good evidence that the activation of CCK-expressing TCs was required for most synaptic excitation of ETd-PG cells.

One potential issue with our results showing that TC suppression reduces evoked excitation of PG cells is the possibility that the effect was an indirect result of TC suppression on MC excitation. Because eTCs are involved in a multistep OSN-to-eTC-to-MC pathway for activating MCs (De Saint Jan et al., 2009; Najac et al., 2011; Gire et al., 2012), suppressing activation of CCK-expressing TCs could reduce MC excitation, which in turn reduces MC-to-PG cell signaling (Najac et al., 2015). We found in a limited number of recordings in MCs that TC inactivation did indeed result in trial-dependent and reversible reduction in MC excitatory current (reduction in integrated charge, 56.52 ± 16.23%; *n *=* *3; *p *=* *0.021, Student’s *t* test; Fig. 4*G–I*), consistent with TC inactivation suppressing MC activation. Thus, the reduced frequency of evoked EPSCs in PG cells during optogenetic suppression of TCs could be explained either because TCs account for the majority of direct excitatory synaptic inputs on PG cells and/or because TC activation is required for MC excitation. Despite this uncertainty, it should be pointed out that, with either scenario, TC activation was a necessary first step in driving most of the excitation of PG cells. Our results explicitly exclude a pathway involving direct inputs from OSNs to MCs (Najac et al., 2011) as a major mechanism of exciting PG cells (i.e., an OSN-to-MC-to-PG cell pathway).

### eTCs provide direct input to PG cells but not eTCs

**Figure 5.**
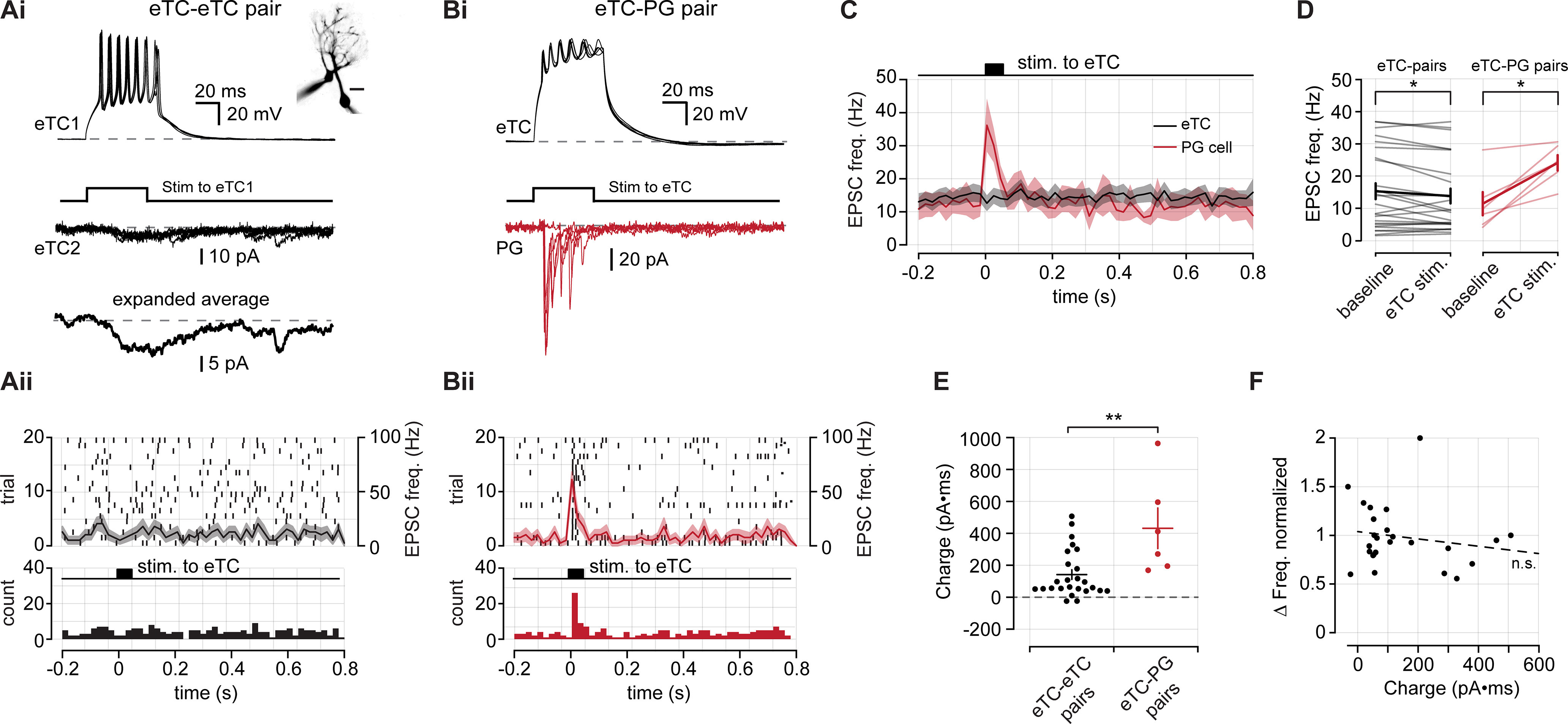
Pair-cell recordings show that eTCs provide direct input to PG cells, but not other eTCs. ***Ai***, Example eTC–eTC pair recording in rat olfactory bulb slices. eTC1 is recorded in current clamp and depolarized to generate spikes. Excitatory currents are recorded from eTC2 in voltage-clamp configuration. Three consecutive sweeps are shown in both cells as well as the average response in eTC2 (bottom). Note that the evoked responses are dominated by a small-amplitude prolonged current. A cell-fill image of the eTC–eTC pair is shown in the inset. Scale bar, 20 μm. ***Aii***, Top, Raster plot of rapid EPSCs detected in eTC2 from ***Ai*** across 20 trials. EPSCs were detected as in previous figures. The black curve is the mean PSTH, and the shaded area is the SEM. Bottom, Pooled bin counts for 20 trials. ***B***, Same as ***A***, but for an eTC–PG cell pair. The eTC was depolarized in current-clamp configuration to generate spikes, and EPSCs were measured from the PG cell in voltage-clamp mode. **C**. Mean rapid EPSC PSTHs from 25 eTC–eTC pairs and 6 eTC–PG cell pairs. ***D***, Summary data of the mean EPSC frequency in the baseline period and the 80 ms following eTC stimulation. ***E***, Summary data comparing the integrated charge in postsynaptic eTCs and PG cells [eTC–eTC pairs (*n *=* *25), 141.65 ± 30.04 pA • ms; vs eTC–PG cell pairs (*n* = 6), 432.49 ± 126.60 pA • ms; *p *=* *0.010, Wilcoxon rank-sum test]. ***F***, Comparison of the normalized change in fast EPSC rate with the magnitude of charge transfer in eTC–eTC pairs (*R* = −0.16; *p *=* *0.44). * denotes *p* < 0.05, ** *p* < 0.01.

Thus far, our optophysiological experiments indicate that CCK-expressing TCs mediate strong excitation of PG cells across a variety of conditions while providing little direct excitatory synaptic input onto eTCs. We next tested, using dual-cell recordings in rat OB slices, whether similar conclusions applied to the presynaptic function of eTCs, which are a subset of CCK-expressing cells. Here, we recorded from one eTC in current-clamp configuration and either another eTC or a PG cell in voltage-clamp mode. We delivered depolarizing current injections to the eTC in current-clamp configuration to generate trains of action potentials (Fig. 5*Ai*,*Aii*). Occasionally, stimulation of an eTC was sufficient to evoke a glomerulus-wide long-lasting depolarization (LLD; Carlson et al., 2000); for our analysis, we did not consider these trials because we could not be certain of the origin of any excitatory signal that we measured during an LLD. In dual-cell recordings in which both cells were eTCs, we found that depolarization of one eTC resulted in small-amplitude but prolonged inward currents (Fig. 5*Ai*). These slow eTC-to-eTC excitatory currents may reflect an extrasynaptic signaling mechanism, as has been described for eTC-to-MC transmission (Gire et al., 2012, 2019), or perhaps weak electrical coupling between eTCs (Hayar et al., 2005; Gire et al., 2012). Importantly, we found no evidence that eTC stimulation evoked fast EPSCs in the eTC–eTC pairs that is consistent with typical synaptic connections (baseline, 15.28 ± 2.46 Hz; vs following eTC stimulation, 13.79 ± 2.30 Hz; *n *=* *25 pairs; *p *=* *0.023, Wilcoxon signed-rank test; Fig. 5*Ai*,*Aii*,*C*,*D*). In contrast, direct stimulation of an eTC in eTC–PG cell pairs caused a large increase in the frequency of fast EPSCs in the PG cell (baseline, 11.40 ± 3.61 Hz; vs stimulation, 24.09 ± 2.40 Hz eTC; *n *=* *6 pairs; *p *=* *0.031, Wilcoxon signed-rank test; Fig. 5*C*,*D*), as expected for direct eTC-to-PG cell contacts (Hayar et al., 2004b; Najac et al., 2015; Tavakoli et al., 2018). Notably, in our analysis of fast EPSCs in the eTC–eTC pairs, we found that direct stimulation of an eTC not only failed to induce fast EPSCs, but in fact, induced a small but significant reduction in the frequency of fast EPSCs (Fig. 5*D*). The cause of the reduced EPSC frequency could be modulation of glutamate release from OSNs (e.g., via GABA_B_ receptors following activation of PG cells; Nickell et al., 1994; Aroniadou-Anderjaska et al., 2000; Wachowiak et al., 2005). Alternatively, it could potentially be an artifact of the higher current noise associated with the evoked slow currents (Fig. 5*Ai*), which impacted the EPSC event detection. We did not further investigate this effect.

The pair-cell recordings provided excellent evidence that eTCs fail to synaptically excite other eTCs, although they can drive a prolonged excitatory current in eTCs. To determine the potential impact of the slow eTC-to-eTC currents, we compared the integrated charge associated with the slow currents with that associated with the more typical barrages of evoked EPSCs in PG cells (Fig. 5*Bi*). To capture the entirety of the slow EPSC, we used a 100 ms integration window that was aligned to the onset of the depolarizing current injection in the presynaptic cell. For both cell types, charge measurements were obtained from composite traces that contained at least 15 trials. We found that for a similar number of presynaptic spikes (eTC–eTC pairs, 4.56 ± 0.33 spikes/trial; eTC–PG cell pairs, 4.43 ± 0.52 spikes/trial; *p *=* *0.940, Wilcoxon rank-sum test) excitation between eTCs is weaker than between eTCs and PG cells [eTC–eTC pairs, 141.65 ± 30.04 pA • ms (*n *=* *25); vs eTC–PG cell pairs, 432.49 ± 126.60 pA • ms (*n *=* *6); *p *=* *0.010, Wilcoxon rank-sum test; Fig. 5*E*]. We did, however, observe a few eTC pairs where the integrated charge was similar to that measured in eTC–PG cell pairs. These instances indicate that under certain conditions, perhaps related to spatial proximity or glutamate clearance properties, eTCs might signal to other eTCs with increased efficacy. We also wondered whether some eTC pairs, those with the largest slow current, displayed evidence for fast synaptic connections (Fig. 5*F*). However, we found no relationship between change in fast EPSC frequency and the magnitude of the slow current (*R* = −0.16, *p *=* *0.44).

### Optical manipulation of intraglomerular inhibition at eTCs

Our studies in CCK-NpHR3.0 mice, together with the pair-cell recordings, provided information about the role of TCs in driving excitation of different cell types in the glomerular layer including PG cells. In the last part of this study, we considered the reverse step: the inhibition of TCs by PG cells. The glomerular layer includes PG cells that differ in expression of GAD isoforms, either GAD65 or GAD67, and also in the number of glomeruli to which they send apical dendrites (Parrish-Aungst et al., 2007; Kiyokage et al., 2010). eTCs can also receive GABAergic input from short-axon cells located in the glomerular layer (Liu et al., 2013; Banerjee et al., 2015). We wondered what contribution GAD65-expressing PG cells have on the inhibition of eTCs. GAD65-expressing PG cells are the most numerous class of PG cells and generally have dendritic arbors that are confined to one glomerulus (Parrish-Aungst et al., 2007; Kiyokage et al., 2010). Hence, they mediate a unique form of local inhibition.

**Figure 6.**
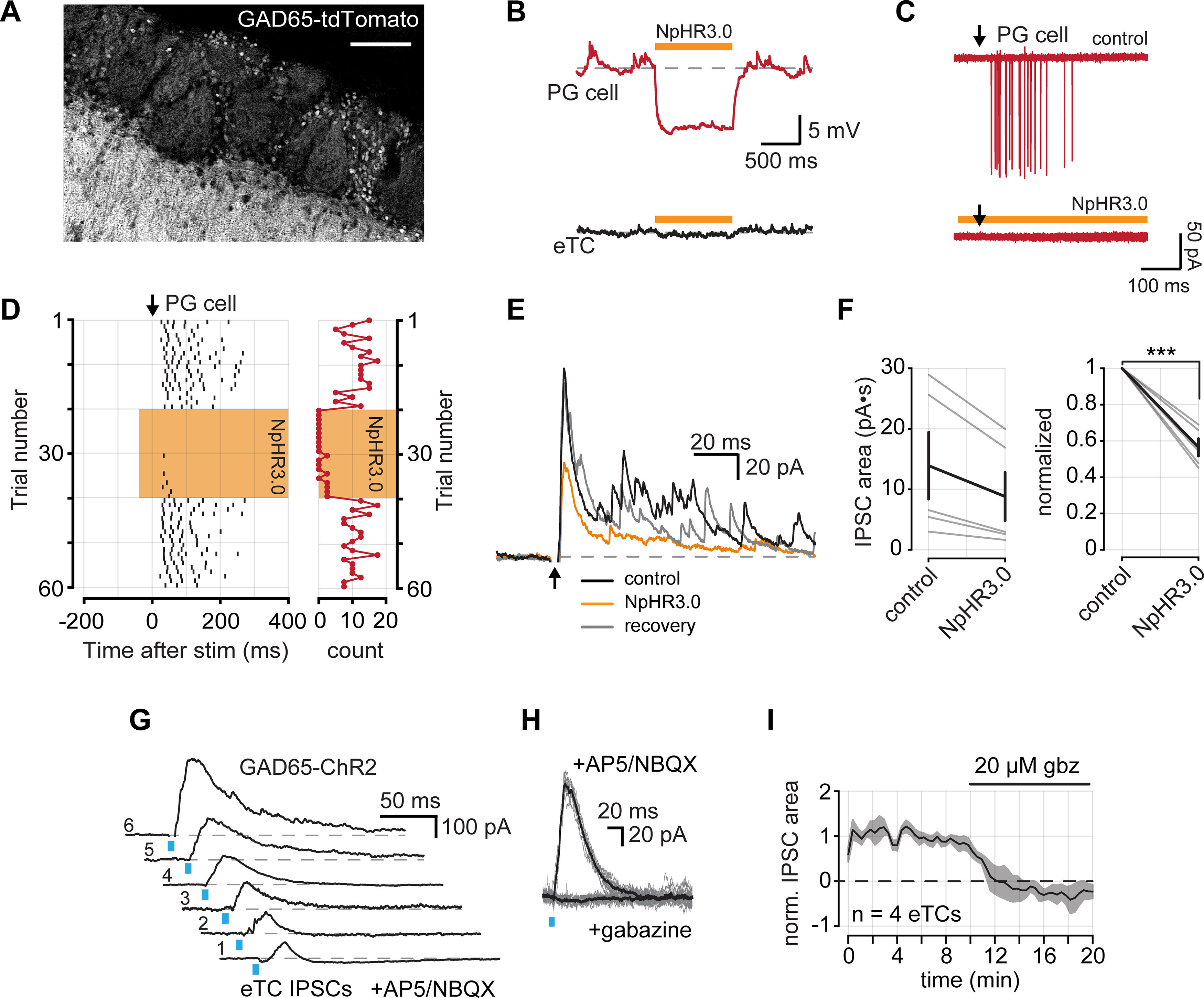
GAD65-expressing PG cells inhibit eTCs. ***A***, GAD65 expression in a tdTomato reporter mouse line. GAD65 is expressed in the soma of PG cells that surround glomeruli. GAD65 expression is also observed in the EPL, likely reflecting granule cell apical dendrites. Scale bar, 100 μm. ***B***, Example membrane voltage responses from a PG cell (top) and an eTC (bottom) in a GAD65-NpHR3.0 mouse in response to a 1 s pulse of 590 nm light. ***C***, Example OSN-evoked (8 μA stimulus) spikes from a PG recorded in LCA configuration. Five overlaid trails under control conditions and during NpHR3.0 activation. Downward arrow corresponds to OSN stimulation. ***D***, Left, Raster plot of evoked spikes per trial from the cell in ***C***. Right, A plot of the number of spikes following each OSN stimulation. ***E***, Example IPSCs evoked by OSN stimulation recorded from an eTC (*V*_hold_ = +28 mV) under control conditions, during NpHR3.0 activation, and following recovery. Each trace is an average of 10 consecutive trials in each condition. ***F***, Left, Summary of OSN-evoked IPSC areas. Right, Summary of normalized IPSC areas (reduction, 43.48 ± 4.76%; *n *=* *5; *p *<* *0.001, Student’s *t* test). ***G***, Example IPSCs recorded from six eTCs following 470 nm LED light stimulation in OB slices prepared from GAD65-ChR2 mice. ***H***, IPSCs were eliminated by bath application of gabazine. Light-colored traces are individual trials and dark is the mean of all trials. ***I***, Time course of IPSC block with gabazine from four eTCs. IPSC areas are normalized to the mean control area for each cell. *** denotes *p* < 0.00.

We generated GAD65-tdTomato mice, in which we confirmed somatic reporter expression interspersed throughout the glomerular layer (Fig. 6*A*), which was consistent with previous reports of GAD65 expression (Parrish-Aungst et al., 2007; Kiyokage et al., 2010; Whitesell et al., 2013; Sun et al., 2020). We also observed dense reporter expression in the EPL that likely arose from the apical dendrites of granule cells that also express GAD65; however, granule cells do not contact eTCs, which lack lateral dendrites. We then generated GAD65-NpHR3.0 mice to selectively manipulate GAD65-expressing cells. NpHR3.0 activation resulted in strong membrane hyperpolarizations in PG cells (Fig. 6*B*) and completely blocked spikes driven by OSN stimulation (Fig. 6*C*,*D*). We next measured the effect of inactivating GAD65-expressing cells while recording OSN-evoked IPSCs in eTCs (*V*_hold_ ≥ 0 mV; Fig. 6*E*). For the current recordings, blocks containing 10 trials with or without light pulses were alternated. When slices were illuminated, the evoked IPSCs in eTCs were reduced by about half (43.48 ± 4.76% reduction in integrated charge; *n *=* *5; *p *<* *0.001, paired *t* test; Fig. 6*F*). However, some IPSCs remained and might reflect either incomplete suppression of activity in GAD65-expressing PG cells or evoked inhibitory input from other GABAergic cell types in the glomerular layer. In four of five recordings, the initial IPSC amplitude was recovered during the second block of control trials (88.76 ± 7.71% of initial control; *n *=* *4), indicating that the reduced IPSC amplitude during the light pulses was not a result of rundown.

As a further confirmation that GAD65-expressing PG cells provide inhibitory input to eTCs, we also measured evoked currents in eTCs from bulb slices prepared from GAD65-ChR2 mice. Recordings were conducted in the presence of the glutamate receptor antagonists NBQX (20 μm) and dl-APV (50 μm) to eliminate the possibility that simultaneously depolarizing a large population of GAD65-expressing cells may have off-target network effects. In each eTC (*n *=* *6) brief light pulses (5 ms at 470 nm) evoked large IPSCs (Fig. 6*G*). We confirmed that these currents were GABA_A_ receptor mediated by their sensitivity to the antagonist gabazine (20 μm; Fig. 6*H*,*I*; *n *=* *4). Together, our findings in GAD65-NpHR3.0 and GAD65-ChR2 mice provide novel optogenetic evidence that local GAD65-expressing PG cells strongly inhibit eTCs.

## Discussion

Prior studies have established that a subset of TCs, the eTCs, play a central role in mediating both inhibition and excitation within OB glomeruli. The inhibition is driven by reciprocal dendrodendritic contacts between eTCs and GABAergic PG cells (Hayar et al., 2004b, 2005; Murphy et al., 2005), while excitation arises from a feedforward pathway wherein OSNs first excite eTCs, which, in turn, excite MCs (OSN-to-eTC-to-MC; De Saint Jan et al., 2009; Najac et al., 2011; Gire et al., 2012). In this study, we used optophysiological tools and pair-cell recordings to delineate several novel aspects of the connectivity between TCs and PG cells that are important for understanding sensory processing in glomerular networks.

### Neural connectivity within olfactory bulb glomeruli

Our first new finding about glomerular connections was that eTCs specifically, and TCs more generally, fail to make chemical synaptic connections onto eTCs. In recordings from eTCs in CCK-NpHR3.0 mice, we found that optogenetically suppressing TCs had no effect on the frequency or amplitude of fast EPSCs that are mediated by typical AMPA receptor glutamatergic connections, both under spontaneous conditions and also following stimulation of OSN axons. If TC-to-eTC connections were prevalent, the suppression of TCs should have reduced the EPSCs in some capacity. The absence of an effect was not because our optogenetic methods were ineffective in suppressing TCs, since our control experiments indicated strong light-evoked suppression of TC output (Fig. 1). The lack of effect was also not because TCs were not active during the conditions of these experiments, since TC suppression was highly effective in reducing EPSCs in PG cells reflecting direct eTC-to-PG cell contacts (Hayar et al., 2004b). Further arguing that eTCs do not make strong connections onto eTCs was that, in pair-cell recordings, eTC stimulation did not drive rapid EPSCs in eTCs, although such stimulation was effective in doing so in eTC–PG cell pairs. Interestingly, we did find in the eTC–eTC pairs that eTC stimulation resulted in a low-amplitude slow excitatory current that lasted ∼100 ms, suggesting that TCs may at a minimum weakly excite eTCs through other modes of signaling. The slow eTC currents resembled slow currents that have been reported in eTC-to-MC pairs that result from the spillover of glutamate at eTC-to-PG cell synapses (Gire et al., 2019), suggesting the possibility of a similar underlying mechanism.

The question of whether there are glutamatergic synaptic connections between TCs/eTCs has been addressed previously using ultrastructural methods. Kosaka and Kosaka (2005) reported the presence of dendrodendritic synaptic connections between excitatory dendrites within glomeruli of mouse OB, potential TC-to-TC contacts, although they did not quantify their number or determine cell types. Prior studies from our laboratory (Bourne and Schoppa, 2017) also provided evidence for such connections in glomeruli of rat OB, although their frequency of occurrence was very low. The evidence from our physiological studies here for negligible TC-to-eTC connections fits well with the latter, more quantitative ultrastructural results, and it is gratifying that the two analysis methods, which rely on different assumptions, led to similar conclusions. An additional point that should be made about the physiological analysis is that it, unlike the ultrastructural work, was agnostic about the location and specific type of glutamatergic connections between eTCs being tested. There is now clear evidence that at least some subtypes of TCs can form local axon collaterals within OB (Ojima et al., 1984; Orona et al., 1984; Igarashi et al., 2012; Sun et al., 2020), which in principle could form axodendritic contacts onto eTCs either within or outside of glomeruli. Our results showing that TCs do not mediate fast EPSCs in eTCs argues against both axodendritic and dendrodendritic contacts between TCs/eTCs. Despite a lack of direct synaptic transmission between eTC pairs, in several instances, we found evidence for prolonged excitatory signaling between eTCs. This finding is supported by another recent physiological study of bulb connectivity (Tavakoli et al., 2018) that also observed evidence for slow prolonged signaling between eTCs (in two pairs).

The second new result from our study regards the previously described dendrodendritic connections that eTCs make onto ETd-PG cells (Hayar et al., 2004b). While it has been unambiguous that these connections exist, it has been uncertain how much these inputs contribute to excitation of PG cells versus other sources such as MCs (Najac et al., 2015). The CCK-NpHR3.0 mice provided a tool to estimate the contribution of CCK-expressing neurons, which, while not restricted to eTCs, are inclusive of eTCs (Fig. 1; Sun et al., 2020). The fact that optogenetic suppression in CCK-NpHR3.0 mice resulted in a 70–75% decrease in the spontaneous and evoked EPSCs in PG cells is consistent with the majority of excitatory inputs into ETd-PG cells reflecting inputs from CCK-expressing TCs under these conditions.

There are some limitations with our results around signaling from TCs onto ETd-PG cells. Perhaps most notable is the fact that CCK is also expressed in the anterior olfactory nucleus (AON; Rothermel et al., 2013), raising the possibility that some of the light-induced reduction in spontaneous EPSCs in CCK-NpHR3.0 mice could have been because of the silencing of AON-to-PG cell projections. However, supporting the conclusion that a large fraction of the silenced sEPSCs reflected inputs from eTCs was the fact that the events often occurred as bursts (Fig. 2*B*). Bursting sEPSCs would be expected if they were derived from eTCs that engage in spontaneous spike bursts (Hayar et al., 2004a; Shao et al., 2009). We cannot exclude the possibility that inputs from AON would also have been bursting in our experiments, but this seems quite unlikely given that any bursting behavior in AON neurons, if it exists, would likely have originated in somatic or dendritic compartments (Raus Balind et al., 2019) that were likely to be largely absent in our slices. Additional support for the conclusion that most of the silenced sEPSCs were derived from eTCs was the fact that silencing NpHR3.0-expressing neurons had no effect on sEPSCs recorded in eTCs (Fig. 2*H*). Because AON cells synapse onto eTCs as well as PG cells (Markopoulos et al., 2012), a mechanism in which the silenced sEPSCs in PG cells reflected AON inputs would require that CCK-expressing projections from AON selectively target PG cells, different from the general population of AON projections. While possible, such a scenario would require an additional level of complexity for which evidence is lacking. A final point, worth emphasizing, is that any uncertainty pertaining to the source of silenced spontaneous EPSCs in PG cells does not extend to our results showing that evoked EPSCs were also reduced by light. It is highly unlikely that, in our slices, the stimulation of olfactory nerve could have resulted in significant activation of feedback fibers onto our test PG cells. Thus, whereas the conclusion that TCs provide the majority of excitatory input into ETd-PG cells requires some qualification when considering the spontaneous condition (because of potential AON input), this is less true for evoked excitation of ETd-PG cells.

A third and final new mechanism pointed to by our optophysiological studies regards the inhibitory synaptic input that eTCs receive from GABAergic interneurons. Prior studies (Parrish-Aungst et al., 2007; Kiyokage et al., 2010) have established that the glomerular layer includes GABAergic PG cells that can be biochemically segregated by the expression of different isoforms of GAD (65 or 67). These biochemically defined PG cells also differ in their anatomy. Most GAD65-expressing PG cells are “uniglomerular,” having an apical dendrite confined to one glomerulus, while most GAD67-expressing PG cells have apical dendrites that extend into multiple glomeruli (Kiyokage et al., 2010) and axons that can extend across the glomerular layer. Based on the fact that suppressing GAD65-positive cells in GAD65-NpHR3.0 mice resulted in a large reduction in evoked IPSCs in eTCs, we conclude that a significant part of the inhibition under this condition was provided by GAD65-positive, uniglomerular PG cells. A broader conclusion can also be made. If we consider the type of PG cells that we were studying in the analysis of TC-to-PG cell connectivity (see above). Because the postsynaptic PG cells in those studies were uniglomerular, our results together argue that there are strong reciprocal connections between TCs and GAD65-positive, uniglomerular PG cells.

Among the caveats in the analysis of inhibition onto eTCs is the fact that the biochemical segregation of PG cells is not precise, with some GAD67-expressing cells coexpressing or transiently expressing GAD65 (Homma et al., 2019). However, the prevalence of these cells is low. There is also evidence that a subpopulation of GABAergic deep short-axon cells (dSACs) make projections to the glomerular layer and form synapses with eTCs (Burton et al., 2017). While these dSACs have not yet been biochemically defined with respect to GAD expression, we cannot exclude the possibility that these cells could have mediated a portion of the IPSC recorded in eTCs.

### Functional implications

What are the functional implications of our new findings about the microcircuitry of glomeruli in OB? If we assume a model in which the output of a glomerulus is determined by the balance between excitation and inhibition, our combined results point toward a scenario in which odors should generally fail to produce a glomerular output because of an unfavorable excitation and inhibition (E/I) balance. We failed to observe synaptic connections among TCs/eTCs, which could underlie recurrent excitation, while, at the same time, eTCs make strong reciprocal connections with a specific class of inhibitory PG cells. Our conclusion that the glomerular E/I balance should generally be weighted toward inhibition is also supported by the available literature characterizing odor-evoked responses in MCs. For example, MCs are much more narrowly tuned to odors than are OSNs (Tan et al., 2010; Kikuta et al., 2013), which at least in part reflects odor-evoked inhibition (Yokoi et al., 1995). It should be pointed out that, in tying our results about the E/I balance at the level of TCs/eTCs to the properties of output MCs, there is an assumption that the E/I balance in TCs/eTCs should impact MC activation. This is well supported by the available literature. Brain slice studies have shown that the majority of the excitatory current in MCs following OSN stimulation is driven by eTCs (Gire et al., 2012; Vaaga and Westbrook, 2016). Thus, the activation status of eTCs, which is dictated by their E/I balance, should be a major contributor to MC activation.

If the E/I balance in a glomerulus generally favors inhibition, might there be conditions in which the E/I balance can become more favorable? Some clue to this was provided by recent studies that analyzed eTC-to-MC feedforward excitation across a range of stimulus conditions (Gire et al., 2019). These studies found that weak stimuli (few spikes in eTCs) produced very little eTC-to-MC excitation, but this excitation rose in a highly supralinear fashion with increasing spike number in the eTC. Such changes, which may in part reflect the dynamics of extrasynaptic glutamate that underlies feedforward excitation, could be critical for allowing MCs to be excited by a select set of “strongest” stimuli, contributing to the narrow tuning of MCs (see above). We suggest that similar mechanisms could be revealed if we were to examine the relationship between increasing spike number in the eTC and the slow excitatory current that we observed here in eTCs. Our preliminary analysis suggested that this slow current had a small magnitude, but a systematic analysis might reveal large eTC-to-eTC currents at high eTC spike number.

Mechanisms that contribute to a more favorable E/I balance under strong stimulus conditions may also extend to those that control the level of GABAergic inhibition. For example, group II metabotropic glutamate receptors (mGluRs) on PG cells are activated by extrasynaptic glutamate from eTCs, leading to a reduction in GABA release from PG cells (Zak et al., 2015). Because an extrasynaptic localization for group II mGluRs might require strong excitation of eTCs to generate glutamate transients sufficient to activate the receptors, reduced GABAergic inhibition—and a more favorable E/I balance—might be specific for conditions of strong OSN activity. Furthermore, pair-cell recordings have indicated thar excitatory signals from eTCs to PG cells depress with increasing number of eTC spikes (Gire et al., 2019). Clearly, more studies are needed to understand eTC-to-eTC signaling under a range of stimulus conditions, as well as how they are balanced with inhibition.

## Synthesis

Reviewing Editor: Christina Zelano, Northwestern University - Chicago Decisions are customarily a result of the Reviewing Editor and the peer reviewers coming together and discussing their recommendations until a consensus is reached. When revisions are invited, a fact-based synthesis statement explaining their decision and outlining what is needed to prepare a revision will be listed below. The following reviewer(s) agreed to reveal their identity: Shawn Burton.

Both reviewers agreed that this is an interesting and impactful study that was well conducted overall. However, the reviewers raised some important concerns, which should be straightforward to address, and are meant to improve the manuscript. See below:

Reviewer 1:

This study was designed to analyze the functional synaptic connections between two subpopulations of juxtaglomerular neurons - external tufted cells (eTCs) and periglomerular (PG) cells in the olfactory bulb (OB) with optophysiological approaches and dual whole cell patch clamp recordings. Understanding the functional organization among these neurons in the OB glomerulus is important to advancing our knowledge on how olfactory signals are processed in the OB glomeruli, the initial site of synaptic integration in the whole olfactory system. While the reciprocal connections between eTCs and PG cells are not surprising due to the presence of previous reports with direct and/or indirect evidence, one novel finding of this study is that TCs do not form chemical synapses with eTCs. The experimental design is straightforward and data are well presented. However, addressing the following concerns may improve the manuscript.

1. CCK is expressed in multiple subpopulations of TCs as acknowledged by the authors and reported by other studies (Seroogy et al, 1985; Liu, et al, 1994; Sun et al, 2020). Thus, it is difficult to determine how much eTC excitation contributes to sEPSCs in PG cells based on optogenetic inhibition of CCKergic TCs in Fig 1-4 since the study of Sun et al. has demonstrated that the superficial TCs with lateral dendrites provide powerful monosynaptic excitation of PG cells. An analysis on the percentage of each subpopulation of TCs expressing pHR3.0-tdTomato in these experiments might be informative.

2. In line 270-271, results are described as “Moreover, in these cells, NpHR3.0-mediated inactivation of TCs did not affect the short-latency early EPSC (Figure 4A, D).” However, the recording traces in Fig 4A show that amplitude of early EPSCs in PG cell 1 is reversibly reduced by optogenetic inhibition of TCs. In addition, a blown-up inset of the data right after ON stimulation (around time 0 s) in Fig 4D may be helpful for comparison of early EPSCs in PG cells between control and NpHR3.0 activation. Otherwise, it is difficult for readers to tell whether there is any difference.

3. The authors found that stimulation of eTCs evoked a weak and long inward current in eTCs (Fig 5Ai) and interpreted this as resulting from an extrasynaptic event due to glutamate spillover without supporting evidence but by citing a reference (line 287). Considering gap junctions between eTCs (Hayar et al. 2005; Kosaka et al, 2005), would this be due to weak electrical synapses as another possibility?

4. For ChR2 stimulation, 470 nm LED was supposed to be used as stated in line 146 and figure legend 6G. However, the text in Results (line 350) indicated that 423 nm light was used.

5. The eTC firing traces in Fig 5Bi look so different from those in Fig 5Ai leading to a question on whether they are from the same population of cells.

Reviewer 2:

OVERVIEW

While glomeruli are the fundamental computational unit of the olfactory bulb, circuitry within and between glomeruli is extremely difficult to dissect, and how glomeruli ultimately shape sensory information from the periphery remains largely unknown. In their article, “Optical manipulations reveal strong reciprocal inhibition but limited recurrent excitation within olfactory bulb glomeruli,” the authors combine single and paired neuron recordings in acute slices of the olfactory bulb with optogenetic perturbations to provide new insight into the glomerular microcircuit. Results of the study compelling show that while glutamatergic external tufted cell (eTC) interneurons provide a substantial amount of fast excitatory synaptic input to local inhibitory periglomerular cell (PGC) interneurons, eTCs do not form canonical synaptic connections with one another, a surprising result given other forms of recurrent excitation known to exist within the glomerulus. Together with the demonstration that a large subset of PGCs (GAD65-Cre-targeted) mediate a large proportion of recurrent eTC inhibition, these results help explain how inhibition regulates glomerular activity and ultimate output from the olfactory bulb. The study been carefully conducted and is of high quality; clarification and further explanation of the points enumerated below, in addition to addressing a significant caveat in genetically targeting eTCs, will further improve the study.

MAJOR ISSUES

1. CCK is expressed in cells all throughout olfactory cortex, in particular including AON. For example, with unilateral viral injection into the olfactory bulb, Rothermel et al. (2013) demonstrated widespread retrograde infection of projection (likely glutamatergic pyramidal) neurons in both ipsilateral and contralateral AON of gene-targeted CCK-IRES-Cre mice. With a constitutive reporter cross, even more AON cells are likely to express NpHR3.0, as well as cells throughout other brain regions centrifugally innervating the olfactory bulb. Given that AON robustly innervates the glomerular layer and provides excitatory input to PGCs (Markopoulos et al., 2012; Boyd et al., 2012), the strong suppression of spontaneous excitatory input to PGCs upon NpHR3.0 photoactivation in the present manuscript could reflect suppression of both TCs and AON terminals (and potentially other centrifugal inputs), depending on the pattern of Cre expression in this particular transgenic CCK-Cre line. Some description of the expression patterns throughout the rest of the brain of these transgenic mice should thus be provided, and, if expression extends to the AON and/or other olfactory cortical regions, this caveat to the sEPSC data needs to be discussed and the conclusion “Under baseline conditions, TCs provide most of the direct excitatory input onto Type II PG cells” (line 197 and elsewhere) amended accordingly. The lack of an effect of NpHR3.0 photoactivation on eTC spontaneous excitatory input (together with the paired eTC-eTC recordings), however, makes a compelling case for the lack of direct eTC/TC-->eTC connections.

MINOR ISSUES

2. Abstract, lines 16-17: As authors note, eTCs are believed to be bulbar interneurons, distinct from projection MCs and TCs. Moreover, many labs have demonstrated that eTCs and projection TCs exhibit distinct physiologies and synaptic profiles (as also noted by the authors). Therefore, while the results provide good evidence that eTCs and TCs collectively (i.e., those targeted with CCK-Cre) do not directly excite eTCs, the results do not indicate whether the same is true for TCs, as this was not tested and cannot be assumed given the differences between eTCs and TCs. In other words, while CCK(+)-->eTC was tested, CCK(+)-->TC was not tested. Therefore, unless additional data is presented, the results should not be generalized to state that “TCs form potent reciprocal synaptic connections with GAD65-expressing uniglomerular PG cells but not other TCs.” The same logic applies to lines 75-76 of the Introduction.

3. Abstract, lines 17-18: the results support the idea that “local inhibition” generally prevails over “recurrent excitation within a glomerulus", but this does not mean “information transfer to downstream cortical regions” is limited, as information involves more than whether or not a neuron spikes. For example, if recurrent excitation dominated you might see runaway spiking in all MCs and TCs, with OSN input to the glomerulus resulting in little additional increase in M/TC spiking, and thus minimal information conveyed. The latter part of this statement is thus quite speculative and unsupported, and should be modified.

4. Introduction, lines 42-43 (also Results lines 265-266, and throughout manuscript): Type I/II PGC terminology was derived from light and electron microscopy histological examination, while Shao et al. functionally defined PGCs as ONd or ETd. This is minor, but important to be precise, as Shao et al. noted that ETd PGCs did receive weaker levels of direct OSN input, and thus might not correspond 1:1 to the histological type II PGCs (consistent with the results of the current study). The authors should clarify that, by the physiological measures being used, they are examining ONd vs. ETd PGCs, which are likely to predominantly correspond to histological type I and II subtypes.

5. Methods, line 109: typo in “MgCl2l"

6. Methods, line 115 “All For” typo

7. Methods, line 134: “uniglomerilar” typo

8. Results, lines 186-187: That MCs did not display significant light-evoked hyperpolarization is encouraging for the use of CCK-Cre lines to selectively manipulate TCs/eTCs and not MCs in future research. However, shouldn’t MCs show some degree of hyperpolarization from: 1) gap junctional coupling with type II MCs/internal TCs that are targeted by CCK-Cre (as shown in your Fig. 1A), 2) gap junctional coupling with middle/superficial TCs (Ma and Lowe, 2010), 3) gap junctional coupling with eTCs (De Saint Jan et al., 2009; Gire et al., 2012), and 4) loss of eTC-mediated feedforward excitation via spontaneous long-lasting depolarizations - similar to the hyperpolarization of MCs observed following NpHR3.0-mediated hyperpolarization of DAT+ cells (Banerjee et al., 2015)? Some analysis or exploration of this is warranted.

9. Results, Fig. 1D,F: Did all eTCs in the CCK-NpHR3.0 mice exhibit such tonic firing with no signs of more typical eTC rhythmic bursting? This might suggest that the constitutive NpHR3.0 expression may be altering the baseline eTC physiology.

10. Results; Given that NpHR3.0-mediated eTC/TC suppression dramatically reduces spontaneous excitatory input to PGCs (Figs. 2,3), it is surprising that NpHR3.0 photoactivation does not have any impact on PGC membrane potential (Fig. 1B,C). Were the current-clamp recordings collected in the presence of GABAAR antagonist?

11. Results, lines 266-267: “Furthermore, in contrast to Type I PG cells, Type II PG cells were defined not to receive direct synaptic input from OSNs.” This was specifically true of ETd PGCs at “minimal effective stimulus intensities", while at higher stimulus intensities, Shao et al. (2009) did observe short-latency, low-jitter excitatory currents consistent with direct (albeit weak) OSN input. The authors’ results thus seem to provide complementary data in agreement with the results of Shao et al. (2009). Highlighting this consensus would, I think, reinforce and strengthen this important finding. Additional analysis of the jitter of the NpHR3.0-resistant early EPSC could also strengthen the finding that these indeed reflect direct, monosynaptic OSN inputs.

12. Results, Fig. 5: Given that eTC-PGC recordings displayed a reliable increase in postsynaptic EPSC frequency with presynaptic firing, it is clear that these pairs had overlapping dendrites within the same glomerulus to support the synaptic connections. Were the eTC-eTC pairs all confirmed to likewise have overlapping dendrites in the same glomerulus, either through morphological examination (as with the example pair in Fig. 5Ai inset) or spontaneous LLD synchrony? If not, could this underlie the heterogenous eTC-eTC charge transfer in Fig. 5E (e.g., same-glomerulus pairs exhibit >200 pAms)? Also, for the eTC-eTC pairs exhibiting the strongest charge transfer, did presynaptic firing evoke more clear EPSCs in the postsynaptic eTC - suggesting that the most-strongly coupled eTCs are synaptically connected while more-weakly coupled eTCs communicate extrasynaptically - or did all pairs consistently exhibit slow currents (as in Fig. 5Ai)?

13. Results, Fig. 6I: typo “20 um gbz” should be “uM"

14. Results/Discussion: Tavakoli et al. (2018) previously reported (albeit in only 2 of 2 pairs tested) that eTCs unidirectionally excite eTCs (“Cluster B-Cluster B”), and while not commented on, the example currents displayed appear to match the slow currents reported in this study. Concordance of these previous findings with the current results should be discussed.

15. Discussion, lines 423-426: Given that GAD65-Cre;reporter crosses label PGCs but also a fairly large proportion of dopaminergic cells (either through transient GAD65 expression during development or through combined GAD65/67 expression) (e.g., Fig. 1 of Homma et al., 2019), the optophysiological results (Fig. 6) cannot be taken as absolute evidence that at least 50% of evoked inhibition in eTCs derives from GAD65-positive cells, as stated. While the results are certainly consistent with strong connectivity between GAD65-positive PGCs and eTCs, a caveat should be included regarding the ‘off-target’ expression in dopaminergic cells that likely include short-axon cells.

16. Discussion, line 454-455: While the authors reasonably suggest that slow eTC-->eTC excitation is likely to display the same supralinear relationship to spike number as slow eTC-->MC excitation, I think further discussion is needed and warranted to explain (even if speculatively) how glomerulus-wide long-lasting depolarization (LLD) events may emerge, in particular given that: 1) MCs do not reciprocally excite eTCs (De Saint Jan et al., 2009), and nor do TCs (this study); 2) a burst of eTC spikes transfers ∼3X as much excitatory charge to PGCs through fast synaptic transmission than to other eTCs through slow currents (this study); and 3) PGCs are generally believed to substantially outnumber eTCs. All of this would seem to suggest that inhibition substantially overpowers excitation within the glomerulus (as the authors generally conclude), and yet LLDs occur robustly and rhythmically throughout the olfactory bulb and can be triggered by a burst of spikes in just a single eTC (De Saint Jan et al., 2009; Gire et al., 2012, 2019; this study).

## Author Response

Reviewer response to en-NWR-0311-21, “Optical manipulations reveal strong reciprocal inhibition but limited recurrent excitation within olfactory bulb glomeruli.”

We thank each of the reviewers for their insightful comments and constructive suggestions. Below, we consider each comment and explain our revision or provide further explanations as requested. In the manuscript file, major changes are highlighted in red text and all line number callouts reference the marked PDF file. For convenience, where applicable, we have pulled text from the manuscript and placed it directly in this document. Our reviewer response is given below each point raised.

Reviewer 1:

This study was designed to analyze the functional synaptic connections between two subpopulations of juxtaglomerular neurons - external tufted cells (eTCs) and periglomerular (PG) cells in the olfactory bulb (OB) with optophysiological approaches and dual whole cell patch clamp recordings. Understanding the functional organization among these neurons in the OB glomerulus is important to advancing our knowledge on how olfactory signals are processed in the OB glomeruli, the initial site of synaptic integration in the whole olfactory system. While the reciprocal connections between eTCs and PG cells are not surprising due to the presence of previous reports with direct and/or indirect evidence, one novel finding of this study is that TCs do not form chemical synapses with eTCs. The experimental design is straightforward and data are well presented. However, addressing the following concerns may improve the manuscript.

Response:

We would like to thank this reviewer for her/his positive comments and constructive suggestions.

1. CCK is expressed in multiple subpopulations of TCs as acknowledged by the authors and reported by other studies (Seroogy et al, 1985; Liu, et al, 1994; Sun et al, 2020). Thus, it is difficult to determine how much eTC excitation contributes to sEPSCs in PG cells based on optogenetic inhibition of CCKergic TCs in Fig 1-4 since the study of Sun et al. has demonstrated that the superficial TCs with lateral dendrites provide powerful monosynaptic excitation of PG cells. An analysis on the percentage of each subpopulation of TCs expressing pHR3.0-tdTomato in these experiments might be informative.

Response:

We agree with the reviewer that it is impossible to determine based on our studies in CCK-NpHR3.0 mice how much eTCs specifically contribute to sEPSCs in PG cells versus other subtypes of TCs that also express CCK. In the prior manuscript, we were careful when discussing optogenetic manipulations to describe TCs in general. However, we also recognize that the reviewer may have been led to believe that we were mainly using the CCK-NpHR3 mice to test the function of eTCs in particular, based on how we constructed our argument for doing the experiments. To avoid any confusion, we now are more clear in both the Results and Discussion sections that the CCK-NpHR3.0 experiments tested the function of CCK-expressing TCs in general and not just eTCs (new text in lines 217-220 and lines 431-433). We also do not believe that this detracts from our study significantly, as learning about how much all CCK-expressing TCs contribute to excitation of PG cells is an interesting question.

The analysis that the reviewer suggests, assessing the percentage of eTCs versus sTCs that express CCK, would be interesting. However, we did not perform this analysis, in part because it would be quite difficult and time-consuming. Definitive information about the TC subtype could only be obtained based on cell fills and analysis of cell morphology (for example assessing whether the cells have lateral dendrites). In addition - and perhaps importantly -- the information obtained from such cell counts would have only modest value toward understanding the question of what part of the PG cell current was due to eTCs versus sTCs. The answer will depend on not just the number of cells, but also the degree to which the cells are spontaneously excited and also excited by OSN stimulation. While eTCs have been reasonably well-characterized in these respects, this is less true for sTCs. While cell counts might be more useful if the fractions of eTCs and sTCs that express CCK were drastically different, this does not appear to be the case based on simple visualization of the images that we acquired (Figure 1A).

2. In line 270-271, results are described as “Moreover, in these cells, NpHR3.0-mediated inactivation of TCs did not affect the short-latency early EPSC (Figure 4A, D).” However, the recording traces in Fig 4A show that amplitude of early EPSCs in PG cell 1 is reversibly reduced by optogenetic inhibition of TCs. In addition, a blown-up inset of the data right after ON stimulation (around time 0 s) in Fig 4D may be helpful for comparison of early EPSCs in PG cells between control and NpHR3.0 activation. Otherwise, it is difficult for readers to tell whether there is any difference.

Response:

As the reviewer suggests, there was some decrease in the early EPSC amplitude in this one experiment with light, but our data set was too incomplete to make a statement about whether light consistently affected the early EPSC amplitude. Thus, we have decided not to say anything in the manuscript about what light is doing to the early EPSC. We now simply point out, referencing Part D of Figure 4, that we saw evidence for an EPSC with onset kinetics consistent with a monosynaptic OSN-EPSC (lines 264-267 and 731-733), as has been reported by Shao and co-workers (2009). The point we had made in our prior manuscript about the OSN-EPSCs was relatively minor, secondary to what light was doing to the more delayed excitatory signal that accounts for the large majority of PG cell excitation. We also decided to remove example PG cell 1 from Figure 4 because the early EPSC and what light may be doing to it is distracting from the main point being addressed.

3. The authors found that stimulation of eTCs evoked a weak and long inward current in eTCs (Fig 5Ai) and interpreted this as resulting from an extrasynaptic event due to glutamate spillover without supporting evidence but by citing a reference (line 287). Considering gap junctions between eTCs (Hayar et al. 2005; Kosaka et al, 2005), would this be due to weak electrical synapses as another possibility?

Response:

The reviewer makes an excellent point. We now mention that the slow current could be due either to extrasynaptic transmission or electrical coupling between eTCs (lines 311-312).

4. For ChR2 stimulation, 470 nm LED was supposed to be used as stated in line 146 and figure legend 6G. However, the text in Results (line 350) indicated that 423 nm light was used.

Response:

The text in the Results is now corrected (in line 377) to reflect that 470 nm light was used for optogenetic stimulation of ChR2-expressing cells. We thank the reviewer for pointing out our prior error.

5. The eTC firing traces in Fig 5Bi look so different from those in Fig 5Ai leading to a question on whether they are from the same population of cells.

Response:

The two example cells were verified to be eTCs by their morphological features identified from cell-fills. Each cell had relatively large cell bodies, had an apical dendrite that filled one glomerulus, and lacked lateral dendrites. These are all properties of eTCs and do not fit with the properties of any other cells that have been characterized in the glomerular layer. Of course, we cannot exclude the possibility that the two cells represent different subtypes of eTCs that are yet to be defined, but this would be quite speculative. As for the differences in spike amplitude in the two cells, this could have arisen if the cells had different numbers of ion channels, for example different expression of K channels that drive an afterhyperpolarization. At least part of the explanation for the difference in these two particular cells may also have been that the current step (50 pA for both cells) in the cell in Figure 5Bi elicited a much larger step depolarization. This can be seen in the voltage traces in 5Ai and 5Bi during the time period that just preceded the first spike. The effect of the larger step depolarization is that the amplitude of the individual spikes (from peak to trough) was smaller. The different amplitude depolarization for a fixed current input size could have reflected differences in input resistance for eTCs (which, in our hands, have varied by as much as a factor of three).

Reviewer 2:

OVERVIEW

While glomeruli are the fundamental computational unit of the olfactory bulb, circuitry within and between glomeruli is extremely difficult to dissect, and how glomeruli ultimately shape sensory information from the periphery remains largely unknown. In their article, “Optical manipulations reveal strong reciprocal inhibition but limited recurrent excitation within olfactory bulb glomeruli,” the authors combine single and paired neuron recordings in acute slices of the olfactory bulb with optogenetic perturbations to provide new insight into the glomerular microcircuit. Results of the study compelling show that while glutamatergic external tufted cell (eTC) interneurons provide a substantial amount of fast excitatory synaptic input to local inhibitory periglomerular cell (PGC) interneurons, eTCs do not form canonical synaptic connections with one another, a surprising result given other forms of recurrent excitation known to exist within the glomerulus. Together with the demonstration that a large subset of PGCs (GAD65-Cre-targeted) mediate a large proportion of recurrent eTC inhibition, these results help explain how inhibition regulates glomerular activity and ultimate output from the olfactory bulb. The study been carefully conducted and is of high quality; clarification and further explanation of the points enumerated below, in addition to addressing a significant caveat in genetically targeting eTCs, will further improve the study.

Response:

We thank Reviewer 2 for her/his positive comments, recommendations, and careful reading of the manuscript.

MAJOR ISSUES

1. CCK is expressed in cells all throughout olfactory cortex, in particular including AON. For example, with unilateral viral injection into the olfactory bulb, Rothermel et al. (2013) demonstrated widespread retrograde infection of projection (likely glutamatergic pyramidal) neurons in both ipsilateral and contralateral AON of gene-targeted CCK-IRES-Cre mice. With a constitutive reporter cross, even more AON cells are likely to express NpHR3.0, as well as cells throughout other brain regions centrifugally innervating the olfactory bulb. Given that AON robustly innervates the glomerular layer and provides excitatory input to PGCs (Markopoulos et al., 2012; Boyd et al., 2012), the strong suppression of spontaneous excitatory input to PGCs upon NpHR3.0 photoactivation in the present manuscript could reflect suppression of both TCs and AON terminals (and potentially other centrifugal inputs), depending on the pattern of Cre expression in this particular transgenic CCK-Cre line. Some description of the expression patterns throughout the rest of the brain of these transgenic mice should thus be provided, and, if expression extends to the AON and/or other olfactory cortical regions, this caveat to the sEPSC data needs to be discussed and the conclusion “Under baseline conditions, TCs provide most of the direct excitatory input onto Type II PG cells” (line 197 and elsewhere) amended accordingly. The lack of an effect of NpHR3.0 photoactivation on eTC spontaneous excitatory input (together with the paired eTC-eTC recordings), however, makes a compelling case for the lack of direct eTC/TC-->eTC connections.

Response:

The reviewer raises an excellent point regarding the source of spontaneous EPSCs in PG cells, which is now discussed with new text in the Discussion section (lines 437-459):

"There are some limitations with our results around signaling from TCs onto ETd-PG cells. Perhaps most notable is the fact that CCK is also expressed in the anterior olfactory nucleus (AON; Rothermel et al., 2013), raising the possibility that some of the light-induced reduction in spontaneous EPSCs in CCK-NpHR3.0 mice could have been due to silencing of AON-to-PG cell projections. However, supporting the conclusion that a large fraction of the silenced sEPSCs reflected inputs from eTCs was the fact that the events often occurred as bursts (Figure 2B). Bursting sEPSCs would be expected if they were derived from eTCs that engage in spontaneous spike bursts (Hayar et al., 2004b; Shao et al., 2009). We cannot exclude the possibility that inputs from AON would also have been bursting in our experiments, but this seems quite unlikely given that any bursting behavior in AON neurons, if it exists, would likely have originated in somatic or dendritic compartments (e.g., Raus Balind et al., 2019) that were largely absent in our slices. Additional support for the conclusion that most of the silenced sEPSCs were derived from eTCs was the fact that silencing NpHR3.0-expressing neurons had no effect on sEPSCs recorded in eTCs (Figure 2H). Because AON cells synapse onto eTCs as well as PG cells (Markopoulos et al., 2012), a mechanism in which the silenced sEPSCs in PG cells reflected AON inputs would require that CCK-expressing projections from AON selectively target PG cells, different from the general population of AON projections. While possible, such a scenario would require an additional level of complexity for which evidence is lacking. A final point, worth emphasizing, is that any uncertainty pertaining to the source of silenced spontaneous EPSCs in PG cells does not extend to our results showing that evoked EPSCs were also reduced by light. It is highly unlikely that, in our slices, stimulation of olfactory nerve could have resulted in significant activation of feedback fibers onto our test PG cells. Thus, whereas the conclusion that TCs provide the majority of excitatory input into ETd-PG cells requires some qualification when considering the spontaneous condition (due to potential AON input), this is less true for evoked excitation of ETd-PG cells.”

Our argument here is essentially three-fold. First, the sEPSCs that we recorded in PG cells often occurred in a burst-like fashion, as would be expected if they were derived from eTCs that are well known to engage in spike bursts. Second, because light did not significantly impact spontaneous EPSCs in eTCs, it is unlikely that the AON projections accounted for a significant fraction of the light-evoked reduction in spontaneous fast EPSCs in PG cells. This point uses the results of Markopoulos and co-workers (2012) that provided unambiguous evidence that AON projection neurons target both PG cells and eTCs. Third, it is very unlikely that the light-evoked reduction of the evoked EPSCs in PG cells could have been explained by AON-to-PG cell connections.

Numerous other changes were also made in the manuscript to address the Reviewer’s concern. For example, we replaced the example PG cell that had been in Figure 2B with a new PG cell that displayed bursting behavior in the sEPSCs. The Results section (lines 222-226) now describes this cell. Moreover, in our rationale for the studies examining ETd-PG cells (e.g., see text in lines 217-220), we no longer imply as we did before that the CCK-NpHR3.0 mice provide a useful tool for teasing out the contribution of bulbar TC inputs to excitation of ETd-PG cells versus centrifugal sources such as AON. While we do not believe that the centrifugal inputs contributed significantly to the sEPSCs in PG cells for the reasons outlined above, we recognize that these mice may be less useful for explicitly addressing that question. Lastly, we have amended the specific sentence mentioned by the Reviewer in the Results (previous line 197) to the following (lines 248-251):

"Overall, our analysis of EPSC amplitudes was consistent with the conclusions from analyzing sEPSC frequency, that TCs are a major source of excitatory input onto ET-PG cells under baseline conditions but they provide little excitatory input onto eTCs (see Discussion).”

The phrase “the major source of excitatory input” has been replaced with “a major source of excitatory input”. Several other changes were also made throughout the manuscript to avoid prior ambiguities.

It should be noted that we have not conducted the additional anatomical analysis that tests for expression of CCK across the whole brain, as requested by the reviewer. However, given the reasonable likelihood that AON cells express CCK in our mice, we have added a significant discussion of the caveat, as requested by the Reviewer (see above), and qualified our results in various parts of the text.

MINOR ISSUES

2. Abstract, lines 16-17: As authors note, eTCs are believed to be bulbar interneurons, distinct from projection MCs and TCs. Moreover, many labs have demonstrated that eTCs and projection TCs exhibit distinct physiologies and synaptic profiles (as also noted by the authors). Therefore, while the results provide good evidence that eTCs and TCs collectively (i.e., those targeted with CCK-Cre) do not directly excite eTCs, the results do not indicate whether the same is true for TCs, as this was not tested and cannot be assumed given the differences between eTCs and TCs. In other words, while CCK(+)-->eTC was tested, CCK(+)-->TC was not tested. Therefore, unless additional data is presented, the results should not be generalized to state that “TCs form potent reciprocal synaptic connections with GAD65-expressing uniglomerular PG cells but not other TCs.” The same logic applies to lines 75-76 of the Introduction.

Response:

We agree with the reviewer. We have edited the sentences in the Abstract (lines 16-19) and Introduction (lines 72-75) to indicate that our results only provide information about TC connections onto eTCs, not TC connections onto TCs broadly.

3. Abstract, lines 17-18: the results support the idea that “local inhibition” generally prevails over “recurrent excitation within a glomerulus", but this does not mean “information transfer to downstream cortical regions” is limited, as information involves more than whether or not a neuron spikes. For example, if recurrent excitation dominated you might see runaway spiking in all MCs and TCs, with OSN input to the glomerulus resulting in little additional increase in M/TC spiking, and thus minimal information conveyed. The latter part of this statement is thus quite speculative and unsupported, and should be modified.

Response:

We have simplified the text and modified our statement to conclude the abstract with, “This configuration favors local inhibition over recurrent excitation within a glomerulus, limiting its output.”

4. Introduction, lines 42-43 (also Results lines 265-266, and throughout manuscript): Type I/II PGC terminology was derived from light and electron microscopy histological examination, while Shao et al. functionally defined PGCs as ONd or ETd. This is minor, but important to be precise, as Shao et al. noted that ETd PGCs did receive weaker levels of direct OSN input, and thus might not correspond 1:1 to the histological type II PGCs (consistent with the results of the current study). The authors should clarify that, by the physiological measures being used, they are examining ONd vs. ETd PGCs, which are likely to predominantly correspond to histological type I and II subtypes.

Response:

We thank the reviewer for pointing this out. The following text (lines 43-46) has been added to clarify the anatomical and physiological distinctions between PG cells. We have also changed Type II to ETd throughout the text to specify the physiological vs. histological distinction.

"These PG cells, which have functionally been defined as eTC-driven (ETd) cells (Shao et al., 2009), account for ∼70% of the total number of PG cells and are likely to predominately correspond to histologically-defined Type II PG cells (Kosaka et al., 1996).”

5. Methods, line 109: typo in “MgCl2l"

Response:

This typo has been corrected. We appreciate this reviewer’s careful reading of our text and methods.

6. Methods, line 115 “All For” typo

Response:

This typo has been corrected.

7. Methods, line 134: “uniglomerilar” typo

Response:

This typo has been corrected.

8. Results, lines 186-187: That MCs did not display significant light-evoked hyperpolarization is encouraging for the use of CCK-Cre lines to selectively manipulate TCs/eTCs and not MCs in future research. However, shouldn’t MCs show some degree of hyperpolarization from: 1) gap junctional coupling with type II MCs/internal TCs that are targeted by CCK-Cre (as shown in your Fig. 1A), 2) gap junctional coupling with middle/superficial TCs (Ma and Lowe, 2010), 3) gap junctional coupling with eTCs (De Saint Jan et al., 2009; Gire et al., 2012), and 4) loss of eTC-mediated feedforward excitation via spontaneous long-lasting depolarizations - similar to the hyperpolarization of MCs observed following NpHR3.0-mediated hyperpolarization of DAT+ cells (Banerjee et al., 2015)? Some analysis or exploration of this is warranted.

Response:

The reviewer is correct to bring up these important considerations for interpreting MC signals upon NpHR3 activation in CCK+ cells. In response, we first point out that we did observe a small 2.1 mV hyperpolarization in one of the three MCs that we tested, even though across the three cells there was no significant hyperpolarization (-1.24 {plus minus} 0.64 mV). We now briefly describe this one MC in the text (lines 196-199), also indicating that it is consistent with prior studies (De Saint Jan et al., 2009; Gire et al., 2012) showing that eTCs and MCs are weakly coupled.

We also expect that the four mechanisms that the reviewer mentions would result in at most a very small light-evoked hyperpolarization in MC. As shown in Figure 1A, expression of CCK appears to be low in MCs, internal tufted cells, and middle tufted cells. While a few of these cells may have expressed CCK, it would be surprising if light-evoked hyperpolarization in a few cells would lead to a large signal in the MC. The eTC-to-MC electrical coupling is arguably the most significant issue, given that CCK appears to be highly expressed in eTCs (Figure 1A) and eTCs are also coupled to MCs (De Saint Jan et al., 2009; Gire et al., 2012). However, the coupling, based on estimated gap junction conductance, is 20-fold lower for eTC-MC pairs versus MC-MC pairs (Gire et al., 2012). As for the last (fourth) mechanism that the reviewer sites involving loss of feedforward excitation, this mechanism would only come into play if a light-evoked hyperpolarization was somehow blocking spontaneously occurring LLDs in the MCs. However, under the conditions of our recordings, LLDs did not spontaneously occur in MCs. Because there are no spontaneous LLDs, there is no significant signal for light-evoked hyperpolarization of eTCs to block in these experiments.

Overall, we believe that our analysis in MCs, while quite limited, fits with reports in the literature. The previously reported weak electrical coupling between eTCs and MCs (and perhaps the coupling involving the other types of TC) predicts that we might observe a weak light-evoked hyperpolarization in the MCs. The fact that we saw a 2.1 mV hyperpolarization in one of the MCs fits with that.

9. Results, Fig. 1D,F: Did all eTCs in the CCK-NpHR3.0 mice exhibit such tonic firing with no signs of more typical eTC rhythmic bursting? This might suggest that the constitutive NpHR3.0 expression may be altering the baseline eTC physiology.

Response:

This is an important consideration. The examples we initially selected here were chosen to highlight the potency of NpHR3.0 in eliminating eTC spiking. Many of the eTCs we recorded from also exhibited the classic theta busting as described by Hayar and others. In Figure 1D we have now replaced the example data with an eTC that showed bursting behavior.

10. Results; Given that NpHR3.0-mediated eTC/TC suppression dramatically reduces spontaneous excitatory input to PGCs (Figs. 2,3), it is surprising that NpHR3.0 photoactivation does not have any impact on PGC membrane potential (Fig. 1B,C). Were the current-clamp recordings collected in the presence of GABAAR antagonist?

Response:

Membrane measurements were made in native recording solution for all cell types. The reason we did not use GABA receptor antagonists is that this often results in glomeruli undergoing frequent spontaneous LLDs which make it difficult to assess NpHR3.0 induced effects.

With respect to PG cell membrane potentials, we did in fact observe small hyperpolarizations in two of six cells of approximately 0.5 mV, consistent with the Reviewer’s proposed mechanism. On the other hand, it is an open question how light-evoked suppression of CCK-expressing TCs would be expected to impact the membrane potential of PG cells, both the magnitude of the effect as well as the directionality. The actual effect will depend on the rate of spontaneous EPSPs in the PG cell, the size of the EPSPs, as well as how EPSPs summate. Furthermore, PG cells receive inhibitory input from other PG cells (Parsa and Vijayaraghavan, 2015) and so light-evoked suppression of eTCs might also reduce IPSPs in the PGs (by inhibiting an eTC-to-PG cell-to-PG cell pathway). Hence, there could be a co-occurring reduction in spontaneous EPSPs and IPSPs. The question posed by the reviewer, while interesting, is complex and beyond the scope of this manuscript. The important point is that PG cells were not significantly hyperpolarized by light, arguing strongly that PG cells do not express CCK that can be activated by light.

11. Results, lines 266-267: “Furthermore, in contrast to Type I PG cells, Type II PG cells were defined not to receive direct synaptic input from OSNs.” This was specifically true of ETd PGCs at “minimal effective stimulus intensities", while at higher stimulus intensities, Shao et al. (2009) did observe short-latency, low-jitter excitatory currents consistent with direct (albeit weak) OSN input. The authors’ results thus seem to provide complementary data in agreement with the results of Shao et al. (2009). Highlighting this consensus would, I think, reinforce and strengthen this important finding. Additional analysis of the jitter of the NpHR3.0-resistant early EPSC could also strengthen the finding that these indeed reflect direct, monosynaptic OSN inputs.

Response:

We thank the Reviewer for pointing this out and for the suggestion to analyze the jitter of the early EPSCs to strengthen our result. After a careful consideration of our data, and issues raised by Reviewer 1, we have determined that our dataset is too incomplete to make any significant claims about OSN inputs to PG cells. Around the issue of jitter, the stimulus intensities we used were modest, which sometimes resulted in failures of the OSN-driven EPSC, thus leading to a relatively small number of trials to analyze and an unreliable estimate of the standard deviation of the onset latency.

We have now simplified the discussion in the Results to point out only that we observed EPSCs in three of six PG cells with onset delays consistent with a monosynaptic EPSC, as has been reported by Shao and co-workers (lines 264-267). Text about these EPSCs is also included in the legend of Figure 4 (lines 731-735). In Figure 4, we have also removed Part J, which had been about the OSN-EPSCs. Our point about OSN-EPSCs in PG cells in the previous manuscript had been secondary and removing these sections avoids distracting from our main point on CCKergic inputs to PG cells.

12. Results, Fig. 5: Given that eTC-PGC recordings displayed a reliable increase in postsynaptic EPSC frequency with presynaptic firing, it is clear that these pairs had overlapping dendrites within the same glomerulus to support the synaptic connections. Were the eTC-eTC pairs all confirmed to likewise have overlapping dendrites in the same glomerulus, either through morphological examination (as with the example pair in Fig. 5Ai inset) or spontaneous LLD synchrony? If not, could this underlie the heterogenous eTC-eTC charge transfer in Fig. 5E (e.g., same-glomerulus pairs exhibit >200 pAms)? Also, for the eTC-eTC pairs exhibiting the strongest charge transfer, did presynaptic firing evoke more clear EPSCs in the postsynaptic eTC - suggesting that the most-strongly coupled eTCs are synaptically connected while more-weakly coupled eTCs communicate extrasynaptically - or did all pairs consistently exhibit slow currents (as in Fig. 5Ai)?

Response:

Each pair of eTCs was visually inspected to confirm their joint association with the same glomerulus. The inspection confirmed that dendrites were overlapping and not confined to separate glomerular sub-compartments. Furthermore, there were only 3/25 pairs where no slow charge transfer was observed, lending confidence that the pairs we recorded from indeed resided within the same glomerulus. We have also added the new text below to the methods (lines 141-146) to make this point clearer.

"In the pair-cell recordings, cell-fill images were used to confirm that the primary dendrite of both neurons occupied the same glomerular neuropil. Visual inspections were further confirmed by measurements of excitatory charge transfer upon stimulation of one of the cells. In 5/5 eTC-PG cell pairs and 22/25 eTC-eTC pairs, stimulation resulted in excitation from one cell to the other, substantiating their joint association with the same glomerulus. In the case of the eTC-eTC pairs, the observed charge transfer was kinetically slow.”

The reviewer makes a good suggestion to characterize the extent of the dendritic overlap with respect to the charge transfer. Unfortunately, this would require z-stacks, which we were not able to collect while recording from two cells. Only a single frame image was collected prior to the start of each recording. Even in the most strongly coupled eTC pairs, we never observed fast (or faster) EPSCs than the slow currents depicted.

Inspired by this comment, we have now included a new Figure 5F where we compare the change in fast EPSC rate with the magnitude of charge transfer in eTC-eTC pairs. Our results indicate there is no relationship between the two parameters - larger charge transfer does not result in more fast EPSCs. This figure panel is described in lines 335-338.

13. Results, Fig. 6I: typo “20 um gbz” should be “uM"

Response:

This has been corrected in the figure image.

14. Results/Discussion: Tavakoli et al. (2018) previously reported (albeit in only 2 of 2 pairs tested) that eTCs unidirectionally excite eTCs (“Cluster B-Cluster B”), and while not commented on, the example currents displayed appear to match the slow currents reported in this study. Concordance of these previous findings with the current results should be discussed.

Response:

The following text has been added (lines 410-412) to reference the work of Tavakoli and colleagues.

"The finding of slow evoked excitatory signaling between eTCs is supported by another recent physiological study of bulb connectivity (Tavakoli et al., 2018) that also observed evidence for such signaling (in two pairs).”

We appreciate the reviewer bringing this oversight to our attention. We cited this paper when discussing eTC-to-PG transmission and now do so for the eTC-to-eTC experiments as well.

15. Discussion, lines 423-426: Given that GAD65-Cre;reporter crosses label PGCs but also a fairly large proportion of dopaminergic cells (either through transient GAD65 expression during development or through combined GAD65/67 expression) (e.g., Fig. 1 of Homma et al., 2019), the optophysiological results (Fig. 6) cannot be taken as absolute evidence that at least 50% of evoked inhibition in eTCs derives from GAD65-positive cells, as stated. While the results are certainly consistent with strong connectivity between GAD65-positive PGCs and eTCs, a caveat should be included regarding the ’off-target’ expression in dopaminergic cells that likely include short-axon cells.

Response:

We have removed language that estimated that 50% of the evoked inhibition arose from GAD65-expressing cells and say “much” of the evoked inhibition. We have also addressed the caveat raised by this reviewer in the text below (new text in lines 463-465).

"The segregation is not precise, with some GAD67-expressing cells co-expressing or transiently expressing GAD65 (Homma et al., 2019), but the prevalence of these cells is relatively low.”

16. Discussion, line 454-455: While the authors reasonably suggest that slow eTC-->eTC excitation is likely to display the same supralinear relationship to spike number as slow eTC-->MC excitation, I think further discussion is needed and warranted to explain (even if speculatively) how glomerulus-wide long-lasting depolarization (LLD) events may emerge, in particular given that: 1) MCs do not reciprocally excite eTCs (De Saint Jan et al., 2009), and nor do TCs (this study); 2) a burst of eTC spikes transfers ∼3X as much excitatory charge to PGCs through fast synaptic transmission than to other eTCs through slow currents (this study); and 3) PGCs are generally believed to substantially outnumber eTCs. All of this would seem to suggest that inhibition substantially overpowers excitation within the glomerulus (as the authors generally conclude), and yet LLDs occur robustly and rhythmically throughout the olfactory bulb and can be triggered by a burst of spikes in just a single eTC (De Saint Jan et al., 2009; Gire et al., 2012, 2019; this study).

Response:

Inspired by this comment, we have significantly expanded the discussion around whether there are conditions under which eTC-to-eTC excitation might be larger than GABAergic inhibition from PG cells (lines 493-514). The new text discusses mechanisms that both enhance eTC-to-eTC excitation and also limit PG cell-mediated inhibition.

"If the E/I balance in a glomerulus generally favors inhibition, might there be conditions in which the E/I balance can become more favorable? Some clue to this was provided by recent studies that analyzed eTC-to-MC feedforward excitation across a range of stimulus conditions (Gire et al., 2019). These studies found that weak stimuli (few spikes in eTCs) produced very little eTC-to-MC excitation, but this excitation rose in a highly supralinear fashion with increasing spike number in the eTC. Such changes, which may in part reflect the dynamics of extrasynaptic glutamate that underlies feedforward excitation, could be critical for allowing MCs to be excited by a select set of “strongest” stimuli, contributing to the narrow tuning of MCs (see above). We suggest that similar mechanisms could be revealed if we were to examine the relationship between increasing spike number in the eTC and the slow excitatory current that we observed here in eTCs. Our preliminary analysis suggested that this slow current had a small magnitude, but a systematic analysis might reveal large eTC-to-eTC currents at high eTC spike number.

Mechanisms that contribute to a more favorable E/I balance under strong stimulus conditions may also extend to those that control the level of GABAergic inhibition. For example, group II metabotropic glutamate receptors (mGluRs) on PG cells are activated by extrasynaptic glutamate from eTCs, leading to a reduction in GABA release from PG cells (Zak et al., 2015). Because an extrasynaptic localization for Group II mGluRs might require strong excitation of eTCs to generate glutamate transients sufficient to activate the receptors, reduced GABAergic inhibition - and a more favorable E/I balance - might be specific for conditions of strong OSN activity. Furthermore, pair-cell recordings have indicated thar excitatory signals from eTCs to PG cells depress with increasing number of eTC spikes (Gire et al., 2019). Clearly, more studies are needed to understand eTC-to-eTC signaling under a range of stimulus conditions, as well as how they are balanced with inhibition.”
